# Oral nirmatrelvir-ritonavir for COVID-19 in higher risk outpatients

**DOI:** 10.1056/NEJMoa2502457

**Published:** 2026-04-23

**Authors:** Christopher C Butler, Andrew D. Pinto, Victoria Harris, Jane Holmes, Najib M Rahman, Lucy Cureton, Gail Hayward, Duncan B Richards, David M Lowe, Joseph F Standing, Judith Breuer, Kerenza Hood, May Ee Png, Stavros Petrou, Jienchi Dorward, Mahendra G Patel, Nicholas PB Thomas, Philip Evans, Nigel D Hart, Bhautesh D Jani, Banafshe Hosseini, Srinivas Murthy, Kerry McBrien, Amanda Condon, Emily G. McDonald, Peter Daley, Michelle Greiver, Bruno R. da Costa, Peter Selby, Peter Jüni, Todd C. Lee, Haolun Shi, Michelle A Detry, Christina T Saunders, Mark Fitzgerald, Nicholas S Berry, Benjamin R Saville, Saye H Khoo, Jonathan S Nguyen-Van-Tam, FD Richard Hobbs, Ly-Mee Yu, Paul Little, for the PANORAMIC Trial and CanTreatCOVID Trial Collaborative Groups

**Affiliations:** 1 Nuffield Department of Primary Care Health Sciences, University of Oxford, Oxford, UK; 2 Upstream Lab, MAP Centre for Urban Health Solutions, Li Ka Shing Knowledge Institute, Unity Health Toronto, Toronto, Ontario, Canada; 3 Department of Family and Community Medicine, St. Michael’s Hospital, Toronto, Ontario, Canada; 4 Department of Family and Community Medicine, Faculty of Medicine, University of Toronto, Toronto, Ontario, Canada; 5 Dalla Lana School of Public Health, University of Toronto, Toronto, Ontario, Canada; 6 Respiratory Trials Unit, Nuffield, Department of Medicine, University of Oxford, Oxford, UK; 7 Oxford National Institute for Health and Care Research Biomedical Research Centre, Oxford, UK; 8 Chinese Academy of Medicine Oxford Institute, University of Oxford, Oxford, UK; 9 Department of Medicine, University of Cambridge, Cambridge, UK; 10 Institute of Immunity and Transplantation, University College London, London, UK; 11 Infection, Inflammation and Immunology, UCL Great Ormond Street Institute of Child Health, London, UK; 12 Centre for Trials Research, Cardiff University, Cardiff, UK; 13 Centre for the AIDS Programme of Research in South Africa (CAPRISA). South Africa; 14 National Institute Health and Care Research Delivery Network, UK; 15 Windrush Medical Practice, Witney, UK; 16 Royal College of General Practitioners, London, UK; 17 Faculty of Health and Life Sciences, University of Exeter, Exeter, UK; 18 School of Medicine, Dentistry and Biomedical Sciences, Queen’s University Belfast, Northern Ireland, UK; 19 General Practice and Primary Care, School of Health and Wellbeing, MVLS, University of Glasgow, Glasgow, UK; 20 Faculty of Medicine, University of British Columbia, Vancouver, British Columbia, Canada; 21 Department of Family Medicine, University of Calgary, Calgary, Alberta, Canada; 22 Department of Community Health Sciences, University of Calgary, Calgary, Alberta, Canada; 23 Department of Family Medicine, Max Rady College of Medicine, Rady Faculty of Health Sciences, University of Manitoba, Winnipeg, Manitoba, Canada; 24 Department of Medicine, Faculty of Medicine and Health Sciences, McGill University, Montréal, Québec, Canada; 25 Memorial University of Newfoundland, St. John’s, Newfoundland and Labrador, Canada; 26 Clinical Trial Service Unit and Epidemiological Studies Unit, Nuffield Department of Population Health, University of Oxford, Oxford, United Kingdom; 27 Department of Statistics and Actuarial Science, Simon Fraser University, Burnaby, British Columbia, Canada; 28 Berry Consultants, Austin, Texas, USA; 29 Department of Biostatistics, Vanderbilt University School of Medicine, Tennessee, USA; 30 Centre for Experimental Therapeutics, University of Liverpool, Liverpool, UK; 31 Lifespan and Population Health Unit, University of Nottingham School of Medicine, Nottingham, UK; 32 Primary Care Research Centre, University of Southampton, Southampton, UK; 33 Department of Family and Community Medicine, North York General Hospital, Toronto, Ontario, Canada

## Abstract

**Background:**

Nirmatrelvir-ritonavir reduced progression to severe SARS-CoV2 infection in unvaccinated high-risk outpatients. The effectiveness of nirmatrelvir-ritonavir in vaccinated populations has yet to be demonstrated.

**Methods:**

In two open-label platform trials (PANORAMIC in the UK and CanTreatCOVID in Canada), community-dwelling adults (aged ≥ 50 or ≥18 with comorbidities) who tested positive for SARS-CoV-2 and were unwell for ≤5 days were randomized to receive either usual care plus nirmatrelvir-ritonavir (300mg/100mg BID x 5 days) or to usual care alone. Primary outcome was all-cause hospitalization or death within 28 days.

**Results:**

From December 8, 2021 to September 30, 2024, 3516 participants in PANORAMIC and 716 in CanTreatCOVID were randomized. Nirmatrelvir–ritonavir did not reduce hospitalization or death ((PANORAMIC: 14/1698 (0.8%) vs 11/1673 (0.7%); adjusted-odds ratio [OR], 1.18; 95% Bayesian credible interval [BCI], 0.55 to 2.62; probability of superiority 0.334, CanTreatCOVID: 2/343 (0.6%) vs 4/324 (1.2%); adjusted-OR, 0.48; 95% BCI, 0.08 to 2.23; probability of superiority 0.830). Viral load was reduced by the end of treatment. 10 serious adverse events were reported for nirmatrelvir-ritonavir in PANORAMIC and 4 in CanTreatCOVID.

**Conclusions:**

In both open-label trials, nirmatrelvir–ritonavir did not reduce the incidence of hospitalization and/or death in vaccinated high-risk patients (National Institute for Health and Care Research, and others; EudraCT Number 2021-005748-31 (PANORAMIC); ClinicalTrials.gov number NCT05614349 (CanTreatCOVID).

Despite vaccination, acquired immunity, and viral evolution, some patients, particularly those at higher-risk, continue to experience protracted illness and admission to hospital because of SARS-CoV-2 infection.^1^ Early treatment with direct-acting antiviral drugs in the community could prevent deterioration, reduce hospital admission, hasten recovery, and reduce viral shedding and transmissibility. Nirmatrelvir-ritonavir^2–5^ that reduced COVID-19 related hospitalization and death over 28 days in high-risk unvaccinated patients in the Evaluation of Protease Inhibition for COVID-19 in High-Risk (EPIC-HR) trial,^6^ leading to recommendation as first-line therapy for outpatients with COVID-19 at the highest risk for progressing to severe disease despite a large number of drug-drug interactions. ^7,8^ In the standard risk outpatients (high risk vaccinated and low risk unvaccinated) EPIC-SR trial, no difference was demonstrated in the time to sustained alleviation of symptoms and no reduction in COVID-19 hospitalization or death.^9^ Observational studies following licensure have been and are being conducted, but all have issues with residual confounding, confounding by indication, and immortal time bias.^6, 10^

Since the EPIC-HR and EPIC-SR studies many more people have been multiply vaccinated and infected naturally so it is unclear whether nirmatrelvir-ritonavir still benefits those at higher risk. The national UK PANORAMIC and the pan-Canadian CanTreatCOVID trials assessed the effectiveness of nirmatrelvir-ritonavir in reducing hospital admissions and/or death, in mostly vaccinated adults in the community with risk factors for serious COVID-19 illness.

## Methods

### Objectives, Patients and Oversight

UK PANORAMIC and Canadian CanTreatCOVID are national, multicentre, primary care, open-label, prospective, platform adaptive clinical trials evaluating antiviral treatment for SARS-CoV-2 in the community.^11^ Interventions assessed in PANORAMIC included molnupiravir^12^ (from December, 2021 to April, 2022) and nirmatrelvir–ritonavir (from April 20, 2022 to March 28, 2024). CanTreatCOVID studied nirmatrelvir-ritonavir between January 16, 2023 and September 30, 2024, and continues to evaluate an anti-oxidant treatment.^13^

Eligible participants were outpatient adults aged 50 years or older (or 18 years or older with relevant comorbidities); had SARS-CoV-2 symptoms for 5 or fewer days and had a positive polymerase chain reaction (PCR) or rapid antigen SARS-CoV-2 test. Exclusions included pregnancy or breastfeeding, of childbearing potential and unwilling to use effective non-hormonal contraception, already taking nirmatrelvir–ritonavir, or had contraindications to nirmatrelvir-ritonavir including taking medication with important drug-drug interactions or those requiring a renal dose adjustment (see both protocols for details at nejm.org).

The UK Medicines and Healthcare products Regulatory Agency and the South Central-Berkshire Research Ethics Committee of the Health Research Authority approved the PANORAMIC trial protocol. The CanTreatCOVID trial was approved by Health Canada and research ethics boards in the participating provinces across Canada. Separate independent trial steering, and data and safety monitoring committees oversaw both trials. An Enhanced Safety Group monitored any required changes in eligibility and adverse effects in a blinded manner. Written informed consent was obtained from all participants or a legal representative. All the data were available to the authors, who vouch for the accuracy and completeness of the data and protocol adherence.

### Randomization and Masking

For PANORAMIC, potentially eligible people were screened, recruited, and enrolled via 65 PANORAMIC General Practice Hubs across the UK. Participants were also recruited online and by telephone by the central trial team. When nirmatrelvir-ritonavir was introduced to the platform, participants eligible for both molnupiravir and nirmatrelvir–ritonavir were randomly assigned (1:1:1) by medical or research professionals to receive nirmatrelvir–ritonavir plus usual care, molnupiravir plus usual care^12^ or usual care alone, and then changed to 1:1 when the molnupiravir arm closed. A secure, web-based system (Spinnaker; version custom built for the PANORAMIC trial; Spiral Software, Wellington, New Zealand) was used for randomization, stratified by age (<50 years vs ≥50 years) and vaccination status (yes vs no).

CanTreatCOVID participants were invited, screened, recruited, and enrolled through public communications, outreach through healthcare settings, provincial COVID hotlines, and community organizations, and randomized on the day of enrolment using a secured interactive web-based system (REDCap Cloud, v.1.7.2, Montréal, Canada), and randomized 1:1 to usual care and the first therapeutic to be evaluated (i.e. nirmatrelvir-ritonavir), Participants were stratified based on age (<65 years vs. older) using varying block sizes.

In both trials, the random sequence of allocation was concealed using central randomization via a web-based system. Post-randomization, participants, along with team members responsible for recruitment, follow-up, and monitoring, were aware of group assignments.

### Procedures

For both trials, participants in the nirmatrelvir–ritonavir group were asked to take nirmatrelvir at a dosage of 300 mg (two 150 mg tablets) with 100 mg ritonavir (one 100 mg tablet) orally twice daily for 5 days. All participants received a trial information booklet. Participant packages containing nirmatrelvir–ritonavir (along with dosing and safety information) were couriered to participants’ home, along with a pregnancy test, if relevant.

In the UK National Health Service (NHS) patients at very high risk were eligible to receive specific treatment from specialist regional COVID-19 clinics.^11^ In Canada, high-risk patients could also receive nirmatrelvir–ritonavir as part of usual care.

Participants completed an online daily diary for 28 days after randomization in PANORAMIC. Non-responders were telephoned on days 7, 14, and 28. In CanTreatCOVID participants completed an online diary for 14 days, with supplemental calls at day 21 and 28 after randomization (see protocols details).

### Virology Sub Study

Participants enrolled in PANORAMIC between September 2022 to October 2023 were offered the opportunity to participate in a virology sub-study involving interval nasopharyngeal SARS-CoV-2 PCR testing in the first 14 days from enrolment (see protocol).

### Outcome Measures

The primary outcome was all-cause, non-elective hospital admission or death within 28 days of randomization. Hospital admission was defined as at least one overnight stay in hospital, or at least one night in a hospital-at-home program (cared for and monitored by hospital clinicians at home after hospital assessment). This outcome was collected from both participants and healthcare system data in PANORMIC, whilst CanTreatCOVID was reported from participants only. Spending time during the day in a hospital emergency department and hospitalizations for elective procedures planned before trial entry were excluded. The primary outcome of the virology sub-study was undetectable viral load at day 7.

Secondary outcomes included early sustained recovery (recovery by day 14 sustained until day 28), which defined as participant reported recovery by day 14 from randomization and remained recovered by day 28. Time to self-reported recovery was defined as the first instance that a participant reported feeling fully recovered from SARS-CoV-2. Other secondary outcomes, including other measures of recovery, contact with health or social services, and new household SARS-CoV-2 infections (PANORAMIC only) are defined in the Statistical Analysis Plans available at NEJM.Org.

### Statistical Analysis

For both trials, the sample size calculation and statistical analysis are detailed in the supplementary materials. PANORAMIC assumed an event rate lower than had been observed event rate in the PRINCIPLE trial^18–22^ at 3% in the usual care. 5300 per arm to ensure a would ensure a reduction to 2% in the intervention arm at 5% level of significance and 90% power (see study Protocols, available at NEJM.Org). However, the sample size in PANORAMIC was revised in conjunction with the trial steering committee in April 2023 due lower than anticipated event rate. With a control event rate of 1.3%, a relative risk of 0.23 (corresponding to an event rate of 0.3% in the intervention arm and which is smaller than the 88% relative risk reduction reported in the EPIC-HR trial^5^), 1438 participants per group would be required to achieve 80% power at a two-sided 5% significance level.

CanTreatCOVID was based on a 5% event rate in usual care and an expected event rate in the treatment arm reduced to 3.3%, 2981 per treatment group to ensure a 5% level of significance and 90% power. However, CanTreatCOVID stopped recruitment, as recommended by the trial steering committee, based slow recruitment and supply of nirmatrelvir-ritonavir was discontinued as of 31 May 2024.

The primary analysis population was defined as all eligible participants who were randomly assigned and analyzed according to allocation. Effect of treatment (and corresponding 95% Bayesian credible intervals (BCIs)) on the primary outcome was estimated using a Bayesian logistic regression model with weakly informative Cauchy priors, adjusting for comorbidity, age, and vaccination status (Adaptive design report). Due to slower than anticipated recruitment because of the drug combinations’ many potential drug-drug interactions and changing epidemiology, there were no interim performed in either trial, so the success threshold remained at 0·975, as prespecified. Sensitivity analyses were also carried out to assess the robustness of the primary outcome analysis due to missing data. Less than 5% of the data for the primary outcome were missing in PANORAMIC, so no multiple imputation was carried out as specified in the SAP. However, multiple imputation was carried out to assess the impact of missing data in CanTreatCOVID. Tipping point analysis was carried out as a post-hoc sensitivity analysis.

Full details and results are available at NEJM.Org.

Other analyses are detailed in the adaptive design report and the trial specific statistical analysis plans (available at NEJM.Org). The virology analysis was also conducted using similar approach for consistency of reporting in this report. Results were consistent with the pre-specified frequentist approach. All analyses of the secondary outcomes were not adjusted for multiplicity, and their credible intervals should not be used to infer definitive treatment effects.

Since PANORAMIC and CanTreatCOVID are pragmatic trials of an authorized approved drug, we adopted a pharmacovigilance strategy. Adverse events (AEs) were not collected in the usual care arm of PANORAMIC; however, CanTreatCOVID collected AEs in both arms (see Protocols available at NEJM.Org). As symptoms of COVID-19 and medication can be difficult to disentangle, symptoms were routinely collected in daily diaries and compared between arms. All analyses were performed in STATA (version 18.0) and R (version 4.2.1).

## Results

### Participants

We screened 126,421 potential participants in PANORAMIC; 51,042 were ineligible (Figure 1). 29,295 underwent randomization between December 8, 2021, and March 28, 2024, with the nirmatrelvir–ritonavir arm opening on April 20, 2022. 1743 were allocated to nirmatrelvir–ritonavir and 1773 usual care alone, 25,779 were randomized to other treatment arms. Medication was started a median of 4 days after symptom onset. In CanTreatCOVID, 1,997 participants were screened, and 880 were ineligible. 721 underwent randomization between January 16, 2023, and September 30, 2024. 358 participants received nirmatrelvir–ritonavir and 358 usual care alone, 5 were randomized to other arms (Figure 1). Medication was started a median of 3 days after symptom onset. Cross over between arms occurred in 8 usual care patients (0.5%) in PANORAMIC and 11 usual care patients (3.1%) in CanTreatCOVID receiving nirmatrelvir–ritonavir post-randomization. Baseline characteristics were well matched between groups overall and within trials separately (Table 1 and Table S9), and largely representative of the potential intend use population, apart from including fewer males and participants of minority ethic origin (Table S8).

### Primary Outcome

There were 14/1698 (0.8%) primary outcome events in the nirmatrelvir-ritonavir group and 11/1673 (0.7%) usual care group in PANORAMIC (Table 2), and 2/343 (0.6%) and 4/324 (1.2%) reported in the CanTreatCOVID, respectively (Table 3). There were no deaths reported during the time the study was recruiting to Nirmatrelvir-ritonavir in either trial. Both demonstrated no statistical difference between the two treatment groups (PANORAMIC: adjusted-odds ratio [OR], 1.18; 95% Bayesian credible interval [BCI], 0.55 to 2.62; probability of superiority 0.334. CanTreatCOVID: adjusted-OR, 0.48; 95% BCI, 0.08 to 2.23; probability of superiority 0.830).

### Secondary Outcomes

Both trials found higher early sustained recovery observed in the nirmatrelvir-ritonavir groups compared to the usual care groups (Table 2 and 3). In PANORAMIC, 33.0% reported early sustained recovery in the nirmatrelvir-ritonavir group compared to 22.1% in the usual care group (adjusted odds ratio, 95% BCI = 1.74 (1.48 to 2.04), and 69.0% vs 53.1% in CanTreatCOVID, respectively (adjusted odds ratio, 95% BCI = 1.99 (1.40 to 2.87)). Time to self-reported recovery was shorter in nirmatrelvir-ritonavir group compared to the usual care in both trials. Other secondary time-to-event outcomes are reported in Tables S10, S11, Figure S1 – S6 for PANORAMIC and Table S16, S17, Figures S11 – S16 for CanTreatCovid in the appendix.

### Subgroup and Sensitivity Analyses

Results from the pre-specified subgroups were similar in PANORAMIC (Figure S7, S8) and CanTreatCOVID trials (Figure S17, S18). Sensitivity analysis of the impact of prior distribution, missing data, and crossovers showed that the results of the primary outcome were robust for both trials. (Table S12, S18 – S20, Figure 9, 10, 19, 20)

### Safety

Most participants (90.4%) in PANORAMIC have experienced adverse events in the nirmatrelvir-ritonavir group, with 9/1743 (0.5%) experiencing serious adverse events (Table 2, and Table S13 – S15). CanTreatCOVID has reported a higher proportion of serious adverse events in the usual care (3.4%) versus 4/358 (1.1%) in nirmatrelvir-ritonavir (Table 3 and Table S21 – S23). There were 242 participants allocated to nirmatrelvir-ritonavir who withdrew from treatment in PANORAMIC, 128 because of side effects, with dysgeusia and/or nausea the commonest single reason (n=99). No participant withdrew due to adverse events in CanTreatCOVID.

### VIROLOGY SUB-STUDY (PANORAMIC only)

In the less intensively sampled cohort, viral load was reduced to below the lower limit of detection (29.2% vs 16.5%, adjusted OR=2.15, 95% BCI=1.37 to 3.44) at day 5, and viral load reduced by 87% in the nirmatrelvir–ritonavir group compared to the usual care group. At day 14 this difference was smaller (Table 2). Results were consistent in the intensively sample group (Appendix Table S10), and Bayesian analysis was consistent with the pre-specified frequentist approach.

## Discussion

The UK (PANORAMIC) and the Canadian (CanTreatCOVID) studies are randomized controlled evaluations of nirmatrelvir–ritonavir for SARS-CoV-2, and report outcomes for predominantly vaccinated adults in the community at increased risk for severe outcomes.

We found no evidence that early treatment with nirmatrelvir-ritonavir reduced the low incidence of hospitalization and/or death in either study and were unable to identify any pre-specified subgroup with compelling evidence of treatment effect.

We found that open-label nirmatrelvir-ritonavir had a shorter self-reported time to recovery and alleviation of all symptoms to no more than a ‘mild problem’. Although serious adverse events were low, most participants reported adverse events, mainly related to taste and gastrointestinal; treatment discontinuation was relatively common. By the end of treatment viral load was lower in the nirmatrelvir-ritonavir group.

In the EPIC-HR trial^6^ unvaccinated patients without prior infection, among those who received treatment within 5 days, 0.8% in the nirmatrelvir–ritonavir group met the primary endpoint of 28-day all-cause hospitalization or death, compared to 6.3% in the placebo group (RRR 88%). EPIC-HR also found a significant 2-day reduction (13 vs 15 days) in median time to sustained symptom alleviation versus placebo (defined as occurring on the first of 4 consecutive days when all targeted symptoms that were scored as moderate or severe at study entry were scored as mild or absent).^15^ Differences in definitions for measures of recovery may partly explain our slightly greater estimates of benefit on recovery.

EPIC-SR assessed efficacy of nirmatrelvir–ritonavir among unvaccinated adults at standard risk (i.e., without an identified risk factor for progression to severe illness) as well as vaccinated adults with one or more risk factors.^9^ EPIC-SR did not demonstrate a significant difference of self-reported time to sustained alleviation of symptoms (12 days vs 13 days, log-rank p-value=0.60).^9^ We found an estimated sustained alleviation of all symptoms from nirmatrelvir-ritonavir treatment, but participants in our trials were on average about 10 years older and more often had co-morbidities compared to those in EPIC-SR.

Both PANORAMIC and CanTreatCOVID were pragmatic trials with applicability to the populations for which nirmatrelvir-ritonavir might be used in countries with already well vaccinated populations. Medication was given a median of between 3 and 4 days from the start of symptoms. We complemented traditional site-based recruitment methods with additional approaches to enrolment, enabling people with SARS-CoV-2 to participate without leaving home, enhancing research equity and adding to generalizability of the findings. We achieved a sample size in excess of that required to detect the effect size found in the EPIC-HR trial.^6^

In contrast to efficacy trials, we conducted an open-label trial, suited to answering pragmatic questions of the effectiveness during routine clinical care because placebos are generally not used in routine care. Such a design facilitates trial conduct and is unlikely to lead to bias with primary outcomes such as hospital admission and/or mortality.^16–18^

^17^However, an open label design does not allow estimation of the contribution of either placebo or nocebo effects to any observed differences in self-reported outcomes such as time to recovery.^18,19^ This is important, as viral load decreased more quickly in the nirmatrelvir-ritonavir arm than the usual care arm by the end of treatment (day 5), implying a mechanism basis for self-reported recovery outcomes.^20^ Interestingly, time to self-reported sustained alleviation was similar in both our open trial and the EPIC-SR placebo-controlled trial,^9^ but with larger estimates compared to the placebo-controlled EPIC-HR trial that used more stringent recovery definitions. Our PRINCIPLE trial, which had a similar open label design, found no meaningful effect for doxycycline^21^, azithromycin^22^, and ivermectin^23^, a trend for harm from colchicine,^24^ and of benefit from inhaled budesonide in a largely unvaccinated population.^25^

There were small differences between PANORAMIC and CanTreatCovid: the median time to recovery differed, CanTreatCOVID started recruitment later than PANORAMIC, and followed participants daily for the first 14 days, so had less data on potential rebound of symptoms.

There were many similar national and single institution led trials during the pandemic, most of which did not recruit sufficient participants to provide clinically useful findings.^26^ Coordinating studies and combining data collected in the national PANORAMIC and CanTreatCOVID trials that used closely matched protocols identifies a route forward for more efficient and collaborative trials for questions of urgent, international public health importance.

## Conclusions

Early treatment with open-label nirmatrelvir–ritonavir for COVID-19 in the community in vaccinated adults at increased risk of poor outcome did not reduce an already low incidence of hospitalization and/or death in these UK and Canadian national randomized trials.

## Supplementary Material

supplement

## Figures and Tables

**A:**
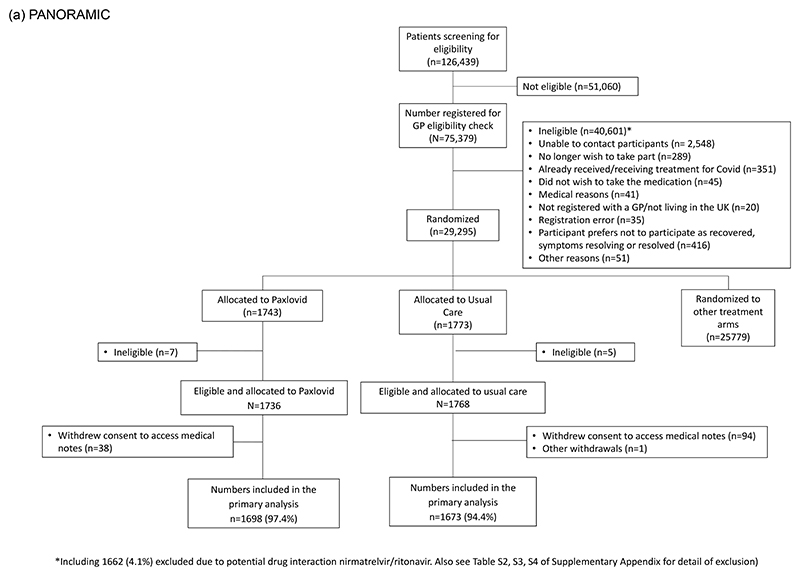


**B:**
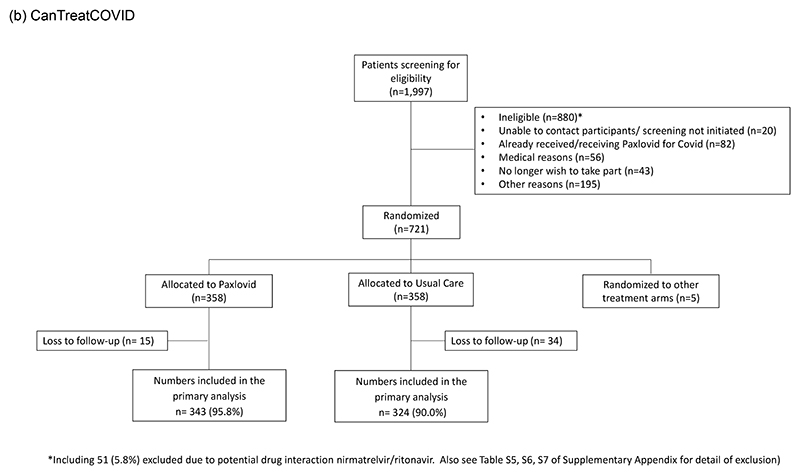


**Table 1:** Baseline characteristics

		PANORAMIC		CanTreatCOVID	
		Nirmatrelvir-ritonavir (N=1736)	Control (N=1768)	Nirmatrelvir-ritonavir (N=358)	Control (N=358)
**Age, mean(SD) [min,max]**		54.7 (12.1) [18 to 96]	54.8 (11.7) [18 to 93]	54.7 (13.6) [19 to 88]	55.0 (13.5) [18 to 89]
Sex*, n(%)	*Female*	1182 (68.1%)	1223 (69.2%)	237 (66.2%)	231 (64.5%)
	*Male*	554 (31.9%)	545 (30.8%)	118 (33%)	100 (27.9%)
	*Other*	0	0	1 (0.3%)	0
	*Missing*	0	0	2 (0.6%)	27 (7.5%)
**Ethnicity category, n(%)**	*White*	1647 (94.9%)	1661 (93.9%)	290 (81%)	272 (76%)
	*Asian*	36 (2.1%)	45 (2.5%)	50 (14%)	36 (10.1%)
	*Mixed Race*	34 (2.0%)	41 (2.3%)	6 (1.7%)	5 (1.4%)
	*Black*	8 (0.5%)	9 (0.5%)	2 (0.6%)	3 (0.8%)
	*Other*	11 (0.6%)	12 (0.7%)	8 (2.2%)	15 (4.2%)
	*Missing, n(%)*	0	0	2 (0.6%)	27 (7.5%)
**Index of Multiple Deprivation (IMD) quintile, n(%)**					
	*(Most deprived) 1*	154 (8.9%)	167 (9.4%)	-	-
	*2*	284 (16.4%)	271 (15.3%)	-	-
	*3*	334 (19.2%)	388 (21.9%)	-	-
	*4*	426 (24.5%)	415 (23.5%)	-	-
	*(Least deprived) 5*	525 (30.2%)	510 (28.8%)	-	-
	*Missing*	13 (0.7%)	17 (1%)	-	-
**Household income**	< *CAD $40,000*			38.0 (10.6%)	46.0 (12.8%)
	≥ *CAD $40,000*			313.0 (87.4%)	280.0 (78.2%)
	*Missing*			7.0 (2.0%)	32.0 (8.9%)
**Duration symptoms at baseline in days, mean(SD) {median (IQR)}**		2.7 (1.2) {3 (2 to 4)}	2.7 (1.2) {3 (2 to 4)}	2.4 (1.1) {2 (2 to 3)}	2.4 (1.1) {2 (2 to 3)}
**Took at least 4 doses IMP, n(%)**		1504 (86.6%)	-	314 (87.7%)	-
	*Missing, n(%)*	74 (4.3%)	-	7 (2%)	-
**Vaccination, n(%)**		1715 (98.8%)	1740 (98.4%)	356 (99.4%)	355 (99.2%)
	*Missing, n(%)*	0	0	0	1 (0.3%)
**Number of vaccine doses, n(%)**	*None*	21 (1.2%)	28 (1.6%)	2 (0.6%)	2 (0.6%)
	*Less than 2*	16 (0.9%)	7 (0.4%)	6 (1.7%)	5 (1.4%)
	*2 or more*	1699 (97.9%)	1733 (98.0%)	350 (97.8%)	350 (97.8%)
	*Missing, n(%)*	0 (0.0%)	0 (0.0%)	0 (0.0%)	1 (0.3%)
**Wellness score t, mean(SD) {median(IQR)}**		4.7 (1.7) {5 (3 to 6)}	4.7 (1.7) {5 (3 to 6)}	-	-
**Comorbidity, n(%)**		1127 (64.9%)	1185 (67.0%)	173 (48.3%)	160 (44.7%)
**Comorbidity- CanTreatCOVID definition**t, **n(%)**		898 (51.7%)	936 (52.9%)	173 (48.3%)	160 (44.7%)

* Self-reported

† Scale from 0 to 10, where 0 represents the worst possible health and 10 the best possible. Note: where no missing category is presented then the data is complete for that variable.

**Table 2:** Primary, Secondary, Safety, and Viral Load Outcomes For Panoramic

	Nirmatrelvir-ritonavir	Usual Care	Estimated Treatment effect (95% BCI)*	Probability of superiority
**Primary outcome**				
Hospitalization or death, n/N (%)	14/1698 (0.8)	11/1673 (0.7)	1.18 (0.55, 2.62) †	0.334
**Secondary outcomes**				
Early sustained recoveryt, n/N (%)	510/1546 (33.0)	330/1492 (22.1)	1.74 (1.48 to 2.04) ‡	
Time to recovery				
Recovered by day 28, n/N (%)	1147/1690 (67.9)	919/1646 (55.8)		
Time to self-reported recovery, median (IQR) §	14 (7 to not reached)	21 (11 to not reached)		
Non-proportional HR (95% BCI) ||				
Day 1 to 2			0.845 (0.390 to 1.796)	
Day 3 to 7			2.123 (1.792 to 2.511)	
Day 8 to 11			1.599 (1.334 to 1.922)	
Day 12 to 28			1.121 (0.987 to 1.271)	
**Adverse events** ¶				
Number of adverse events	4094	-		
Number of participants with at least one adverse events	1575/1743 (90.4%)	-		
**Serious Adverse events**				
Number of serious adverse events	9	-		
Number of participants with at least one serious adverse events	9/1743 (0.5%)	-		
**Virology outcome – all sample cohort**				
Viral load below detection level				
Day 1, n/N (%)	13/330 (3.9)	18/304 (5.9)		
Day 5, n/N (%)	78/267(29.2)	36/218 (16.5)	2.15 (1.37 to 3.44) **	
Day 14, n/N (%)	131/183 (71.6)	106/156 (67.9)	1.30 (0.77 to 2.15) **	
Viral load				
Day 1, geometric mean (SD)	1988856.5 (51.2)	1713635.8 (47.5)		
Day 5, geometric mean (SD)	3587.0 (26.6)	30267.1 (52.3)	0.13 (0.08 to 0.21) ††	
Day 14, geometric mean (SD)	288.7 (9.5)	314.0 (9.0)	0.93 (0.51 to 1.68) ††	

* All credible interval widths for the secondary and virology outcomes have not been adjusted for multiplicity and cannot be used to infer definitive treatment effects.

† Adjusted odds ratio (OR) obtained from Bayesian logistic regression model adjusted for age, vaccination status, and comorbidity at baseline, with 95% Bayesian credible interval (BCI). Odds Ratio < 1 favours nirmatrelvir-ritonavir. Pr(Superiority) is the probability of superiority and treatment superiority is declared if Pr(superiority) ≥ 0·975 versus usual care.

‡ Binary outcome defined as recovered by day 14 with no subsequent instances of “not recovered” until day 28. Adjusted OR obtained from Bayesian logistic regression model adjusted for age, vaccination status, and comorbidity at baseline. Odds Ratio > 1 favours nirmatrelvir-ritonavir.

§ Kaplan-Meier estimates of median time to event and interquartile range from the raw data.

|| Hazard ratio (95% credible interval) for each time interval using a Bayesian time varying piecewise exponential model, adjusting for age, vaccination status and comorbidity at baseline. Time intervals were chosen based on information from a clinician without knowledge of the data.

¶ Did not routinely collect adverse events in the usual care arm.

** Bayesian logistic regression adjusting for sex, age, and baseline log10(viral load). Adjusted OR > 1 favours nirmatrelvir-ritonavir.

†† Bayesian mixed effect model for log_10_(viral load) adjusting for sex, age, and baseline log_10_(viral load); Adjusted geometric mean ratio < 1 favours nirmatrelvir-ritonavir.

**Table 3:** Primary, Secondary, Safety, and Viral Load Outcomes For Cantreatcovid

Outcome	Nirmatrelvir-ritonavir	Usual Care	Estimated Treatment effect (95% BCI) *	Probability of superiority
**Primary outcome**				
Hospitalization or death, n/N (%)	2/343 (0.6)	4/324 (1.2)	0.48 (0.08 to 2.23) †	0.830
**Secondary outcomes**				
Early sustained recovery^†^, n/N (%)	191/277 (69.0)	130/245 (53.1)	1.99 (1.40 to 2.87) ‡	
Time to recovery				
Recovered by day 14, n/N (%)	272/345 (78.8)	194/306 (63.4)		
Time to self-reported recovery, median (IQR) §	6 (4 to 11)	9 (4 to not reached)		
Non-proportional HR (95% BCI)				
Day 1 to 2			0.94 (0.55 to 1.53)	
Day 3 to 7			1.72 (1.35 to 2.23)	
Day 8 to 11			1.54 (1.06 to 2.26)	
Day 12 to 14			1.07 (0.58 to 1.90)	
**Adverse events**				
Number of adverse events	190	38		
Number of participants with at least one adverse event, n/N (%)	112/358 (32.1)	20/358 (5.6)		
**Serious Adverse events**				
Number of serious adverse events	7	16		
Number of participants with at least one serious adverse event, n/N (%)	4/358 (1.1)	12/358 (3.4)		

* All credible interval widths for the secondary outcomes have not been adjusted for multiplicity and cannot be used to infer definitive treatment effects.

† Adjusted odds ratio (OR) obtained from Bayesian logistic regression model adjusted for age, vaccination status, and comorbidity at baseline, with 95% Bayesian credible interval (BCI). Odds Ratio < 1 favours nirmatrelvir-ritonavir. Pr(Superiority) is the probability of superiority and treatment superiority is declared if Pr(superiority) ≥ 0·975 versus usual care.

‡ Binary outcome defined as recovered by day 14 with no subsequent instances of “not recovered”. It is assumed that if the participant was recovered on both day 21 and day 28 calls that they remained recovered. Adjusted OR obtained from Bayesian logistic regression model adjusted for age, vaccination status, and comorbidity at baseline. Odds Ratio > 1 favours nirmatrelvir-ritonavir.

§ Kaplan-Meier estimates of median time to event and interquartile range from the raw data.

|| Hazard ratio (95% credible interval) for each time interval using a Bayesian time varying piecewise exponential model, adjusting for age, vaccination status and comorbidity at baseline. Time intervals were chosen based on information from a clinician without knowledge of the data.
