# Supplementary material for: Oral nirmatrelvir-ritonavir for COVID-19 in higher risk outpatients
Source: N Engl J Med. Author manuscript; Available in PMC 2026 Jun 15. (PMC7619176; doi:10.1056/NEJMoa2502457)
Supplement: supplement [file EMS212211-supplement-supplement.pdf]

Oral Nirmatrelvir-ritonavir plus usual care versus usual care alone as early treatment for adults with SARS-CoV-2 at increased risk of adverse outcomes

Supplementary appendix

Contents

PANORAMIC Trial Collaborative Group 5

CanTreatCOVID Collaborative Group: 9

Listing of comorbidities for inclusion 13

PANORAMIC 13

CanTreatCOVID 13

Virology sub study 14

Sample size determination 14

Adverse event reporting 15

Sample size reconsiderations for the PANORAMIC trial 15

Recruitment overlaps in PANORAMIC 16

Early termination of recruitment in CanTreatCOVID 16

Reasons for not eligible at screening stage 17

PANORAMIC 17

CanTreatCOVID 20

Representativeness of Study Populations 21

Supplementary Tables and Figures for the PANORAMIC 22

Analysis of Individual Symptoms 31

Subgroup Analysis 33

Sensitivity Analysis 35

Exploring the assumptions about the prior distribution 35

NB: All credible interval widths presented in the graphs have not been adjusted for multiplicity and cannot be used to infer definitive treatment effect. 35

Missing Data 35

Adverse Events and Serious Adverse Events 37

Supplementary Tables and Figures for the CanTreatCOVID study 40

Health and social care service use 41

Kaplan-Meier Plots for time to event outcomes 42

Analysis of Individual Symptoms 43

Subgroup Analysis 45

Sensitivity Analysis 47

Exploring the assumptions about the prior distribution 47

Missing Data 47

Adverse Events and Serious Adverse Events 51

List of Supplementary Tables

Table S1 Power and sample size estimates for PANORAMIC per treatment arm based on 33% relative reduction 15
Table S2 Reasons for not eligible from patients online for eligibility (n=51,042) (can be more than one reasons) [PANORAMIC] 17
Table S3 Reasons for not eligible assessed by GP (n=42,137) [PANORAMIC] 18
Table S4 List of medication that patients were not eligible due to interaction with nirmatrelvir–ritonavir (Can have more than one medications) [PANORAMIC] 19
Table S5 Reasons for not eligible from patients self-assessed online - can have multiple reasons (n=21,100) [CanTreatCOVID] 20
Table S6 Reasons for not eligible from study team assessment (n=1,276) [CanTreatCOVID] 20
Table S7 List of medication that patients were not eligible due to interaction with Paxlovid (can have more than one medications) [CanTreatCOVID] 20
Table S8 Summary of generalizability of findings for PANORAMIC and CanTreatCOVID 21
Table S9 Symptoms and comorbidities at baseline [PANORAMIC] 22
Table S10 Secondary outcomes [PANORAMIC] 25
Table S11 Self-reported contacts with healthcare services [PANORAMIC] 28
Table S12 Maximum likelihood model estimates to explore sensitivity to prior distribution [PANORAMIC] 35
Table S13 Adverse events coded by MedDRA Preferred Terms (some AEs have more than one MedDRA codes)* [PANORAMIC] 37
Table S14 Adverse events coded by MedDRA System Organ Class (some AEs have more than one MedDRA codes)* [PANORAMIC] 39
Table S15 Serious adverse events coded by MedDRA System Organ Class [PANORAMIC] 39
Table S16 Secondary outcomes [CanTreatCOVID] 40
Table S17 Self-reported contacts with healthcare services [CanTreatCOVID] 41
Table S18 Maximum likelihood model estimates to explore sensitivity to prior distribution [CanTreatCOVID] 47
Table S19 Multiple imputation results for the primary outcome [CanTreatCOVID] 48
Table S20 Summary of sensitivity analysis of primary outcome [CanTreatCOVID] 49
Table S21 Adverse events coded by MedDRA Preferred Terms (some AEs have more than one MedDRA codes) [CanTreatCOVID] 51
Table S22 Adverse events coded by MedDRA System Organ Class (some AEs have more than one MedDRA codes) [CanTreatCOVID] 53
Table S23 Serious adverse events coded by MedDRA System Organ Class [CanTreatCOVID] 53

List of Supplementary Figures

Figure S1 Kaplan-Meier curves for time to recovery [PANORAMIC] 30
Figure S2 Kaplan-Meier curves for time to sustained recovery [PANORAMIC] 30
Figure S3 Time to alleviation of symptoms [PANORAMIC] 31
Figure S4 Time to sustained alleviation of symptoms [PANORAMIC] 31
Figure S5 Time to reduction of symptoms [PANORAMIC] 32
Figure S6 Recurrence of any symptom [PANORAMIC] 32
Figure S7 Forest plot of Subgroup analysis of primary outcome [PANORAMIC] 33
Figure S8 Forest plot of subgroup analysis of time to first reported recovery [PANORAMIC] 34
Figure S9 Heatplot of the odds ratio compared to different numbers of events within the unobserved data [PANORAMIC] 36
Figure S10 Heatplot of the probability of superiority compared to the number of events within the unobserved data [PANORAMIC] 36
Figure S11 Kaplan-Meier curves for time to recovery [CanTreatCOVID] 42
Figure S12 Kaplan-Meier curves for time to sustained recovery [CanTreatCOVID] 42
Figure S13 Time to alleviation of symptoms [CanTreatCOVID] 43
Figure S14 Time to sustained alleviation of symptoms [CanTreatCOVID] 43
Figure S15 Time to reduction of symptoms [CanTreatCOVID] 44
Figure S16 Time to reduction of severity of symptoms [CanTreatCOVID] 44
Figure S17 Forest plot of Subgroup analysis of primary outcome [CanTreatCOVID] 45
Figure S18 Forest plot of subgroup analysis of time to first reported recovery [CanTreatCOVID] 46
Figure S19 Heatplot of the odds ratio compared to different numbers of events within the unobserved data [CanTreatCOVID] 50
Figure S20 Heatplot of the probability of superiority compared to the number of events within the unobserved data 50

PANORAMIC Trial Collaborative Group

Prof Christopher C Butler, FMedSci[1], Prof F D Richard Hobbs, FMedSci[1], Oghenekome A Gbinigie, DPhil[1], Prof Najib M Rahman, DPhil[2,3,5], Gail Hayward, DPhil[1], Prof Duncan B Richards, DM[4], Jienchi Dorward, MBChB[1,6], David M Lowe, PhD[7], Prof Joseph F Standing, PhD[8,9], Prof Judith Breuer, MD[8], Prof Saye Khoo, FRCP[10], Prof Stavros Petrou, PhD[1], Prof Kerenza Hood, PhD[11], Prof Jonathan S Nguyen-Van-Tam, FMedSci[13], Prof Mahendra G Patel, PhD[1], Benjamin R Saville, PhD[14,15], Joe Marion, PhD[14], Prof Nick Francis, PhD[16], Nicholas P B Thomas, FRCGP[17,18,19], Prof Philip Evans, FRCGP[20,21], Melissa Dobson, BSc[2], Jane Holmes, PhD[1], Victoria Harris, PhD[1], May Ee Png, PhD[1], Mark Lown, PhD[16], Oliver van Hecke, DPhil[1], Michelle A Detry, PhD[14], Christina T Saunders, PhD[14], Mark Fitzgerald, PhD[14], Nicholas S Berry, PhD[14], Sam Mort, PGCert[1], Bhautesh D Jani, PhD[22], Prof Nigel D Hart, MD[23], Haroon Ahmed, PhD[12], Daniel Butler, MBChB[23], Micheal McKenna, BSc[1], Lucy Cureton, BSc[1], Meena Patil[1], Monique Andersson, MD[24], Clare Bateman, BA[1], Jennifer C Davies, PhD[1], Prof Andrew Ustianowski, PhD[25], Prof Andrew Carson Stevens, PhD[12], Ly-Mee Yu, DPhil[1], Prof Paul Little, FMedSci[16]

List of affiliations

1. Nuffield Department of Primary Care Health Sciences, University of Oxford, Oxford, UK
2. Oxford Respiratory Trials Unit, Nuffield Department of Medicine, University of Oxford, Oxford, UK
3. Chinese Academy of Medical Sciences Oxford Institute, University of Oxford, Oxford, UK
4. Nuffield Department of Orthopaedics, Rheumatology and Musculoskeletal Sciences, University of Oxford, Oxford, UK
5. Oxford National Institute for Health and Care Research Biomedical Research Centre, Oxford, UK
6. Centre for the AIDS Programme of Research in South Africa (CAPRISA), University of KwaZulu–Natal, Durban, South Africa
7. Institute of Immunity and Transplantation, University College London, London, UK
8. Infection, Inflammation and Immunology, UCL Great Ormond Street Institute of Child Health, London, UK
9. Department of Pharmacy, Great Ormond Street Hospital for Children, London, UK
10. Department of Pharmacology, University of Liverpool, Liverpool, UK
11. Centre for Trials Research, Cardiff University, Cardiff, UK
12. Division of Population Medicine, Cardiff University, Cardiff, UK
13. Lifespan and Population Health Unit, University of Nottingham School of Medicine, Nottingham, UK
14. Berry Consultants, Austin, TX, USA
15. Department of Biostatistics, Vanderbilt School of Medicine, Nashville, TN, USA
16. Primary Care Research Centre, University of Southampton, Southampton, UK
17. Windrush Medical Practice, Witney, UK
18. National Institute for Health and Care Research Clinical Research Network: Thames Valley and South Midlands, Oxford, UK
19. Royal College of General Practitioners, London, UK
20. Faculty of Health and Life Sciences, University of Exeter, Exeter, UK
21. National Institute for Health and Care Research Clinical Research Network, Leeds, UK
22. General Practice and Primary Care, School of Health and Wellbeing, College of Medical, Veterinary & Life Sciences, University of Glasgow, Glasgow, UK
23. School of Medicine, Dentistry and Biomedical Sciences, Queen's University Belfast, Belfast, UK
24. Department of Microbiology, Oxford University Hospitals NHS Foundation Trust, Oxford, UK
25. Regional Infectious Diseases Unit, North Manchester General Hospital, Manchester, UK

Clinical Safety Call team

Debby Nicoll	Adrian Burns	Florence Conneh
Leon Dong	Mary Green	Bhumika Patel
Amrita Kafle	Andrew Ting	Haroen Sahak
Juliah Jonasi	Wahida Kayyum	Sanjay Ramakrishnan
Rita Corser	Tsvetely Angelova-Cooper	Bindhu Xavier
Darren Smith	Dawn Beaumont-Jewell	Robin Williams
Kerry Goodsell	Samantha Edwards	Chisomo Chitedze
Karolina Krassowska	Jess Trigg	

Non-clinical safety call team

Nicholas Symons	Suzie Engela	Amber Madden-Nadeau
Faith Fordham		

Clinical Safety Monitor

Usha Sukumaran	Areej Moftah

Clinical Safety Monitor (rota cover)

Liliana Cifuentes Gutierrez	Kome Gbinigie	Monique Andersson
Najib Rahman	Chris Turnbull	Rob Hallifax
Anand Sundaralingam		

Independent SAE Reviewers

Mark Lown	Beenish Iqbal	Dinesh Addala
Mark Roberts	Peter Saunders	

Virology Team

Akosua A Agyeman	Divya Shah	Julianne Brown
Chris Thalasselis	Maximillian N J Woodall	Francis Yongblah

Principal investigators and Associate Principal Investigators

Tanveer Ahmed
Damien Allcock
George Atherton
Oluseye Emmanuel Benedict
Adrian Beltran-Martinez
Nigel Bird
Gerard Burns
Laura Brennan
Mike Butler
Daniel Butler
Andrew Carson-Stevens
Zelda Cheng
Ruth Danson
Nigel de Kare-Silver
Devesh Dhasmana
Jon Dickson
Serge Engamba
Stacey Fisher
Robin Fox
Eve Frost
Richard Gaunt
Sarit Ghosh
Ishtiaq Gilkar
Anna Goodman
Steve Granier
Aleksandra Howell
Iqbal Hussain
Simon Hutchinson
Marie Imlach
Greg Irving
Nicholas Jacobsen
James Kennard
Umar Khan
Kyle Knox
Christopher Krasucki
Tom Law
Rem Lee
Nicola Lester
David Lewis
James Lunn
Claire I. Mackintosh
Mehul Mathukia
Patrick Moore
Seb Morton
Daniel Murphy
Rhiannon Nally
Chinonso Ndukauba
Olufunto Ogundapo
Henry Okeke
Amit Patel
Kavil Patel
Ruth Penfold
Satveer Poonian
Olajide Popoola
Alexander Pora
Vibhore Prasad
Rishabh Prasad
Omair Razzaq
Scot Richardson
Simon Royal
Afsana Safa
Satash Sehdev
Tamsin Sevenoaks
Aadil Sheikh
Vanessa Short
Baljinder Singh Sidhu
Ivor Singh
Yusuf Soni
Pete Wilson
David Wingfield
Michael Wong
Nick Wooding
Sharon Woods
Joanna Yong
Azhar Zafar

Data and Safety Monitoring Committee Independent members

Prof Deborah Ashby (Chair) Prof Simon Gates Prof Benjamin Fisher

Prof Gordon Taylor Prof Martin Underwood

Trial Steering Committee Independent members

Prof Philip Hannaford (Chair) Ms Corina Cheeks Prof Ranjit Lall

Prof Alastair Hay Prof William Hollingworth

CanTreatCOVID Collaborative Group:

Andrew Pinto, MD [1,2,3,4], Benita (Banafshe) Hosseini, PhD[1,3,4], Amanda Condon, MD[5], Bruno da Costa, PhD [4,6], Peter Daley, MD[7], Michelle Greiver, MD[3], Peter Jüni, MD[4,6], Todd C. Lee, MD[8], Kerry McBrien, MD[9,10], Emily McDonald, MD[8], Srinivas Murthy, MD[11], Peter Selby, MBBS[3,4,12], Haolun Shi, PhD [13], Melissa Andrew, MD[14], Kris Aubrey-Bassler, MD[7], David Barber, MD[15], Brendan Barrett, MB[7], Mylaine Breton, PhD [16], Christopher C Butler, MD[17], Noah Crampton, MD[3,18], Simone Dahrouge, PhD [19,20,] Ali Damji, MD[3,21], Robert Fowler, MDCM[3,22], Stephanie Garies, PhD [1,2], Catherine Hudon, MD[23], Jennifer Hulme, MDCM[3,18], Jennifer Isenor, PharmD[24], Aisha Lofters, MD[1,3,25], David Jenkins, MD[1,3,26,27], Rosemarie Lall, MD[3,28], Annie LeBlanc, PhD [29, 30], Christine Leong, PharmD[31,32], Paul Little, FMedSci[33], Sarvesh Logsetty, MD[31,34], Sylvain Lother, MD[5], Marie-Thérèse Lussier, MD[35], Laura MacLaren, PhD [8], Derelie Mangin, MBChB[36], Emily Marshall, PhD [14], John Marshall, MD[37], Rita McCracken, MD[11], Rahim Moineddin, PhD [3], Briana Orava, PhD [38], Jean-Sebastien Paquette, MD[29,30], Jay Jae Hee Park, PhD [39], Navindra Persaud, MD[1,2,3,4], Valeria Rac, MD[4,18], Vivian Ramsden, PhD [40], Jennifer Rayner, PhD [41,42], Diana Sanchez Ramirez, PhD [43], Lynora Saxinger, MD[44], Alexander Singer, MB BAO BCh [5], Rae Spiwak, PhD [33], Anita Srivastava, MD[3,45], Abhimanyu Sud, MD[3,46], Jean-Éric Tarride, PhD [37], Deanna Telner, MD[3], Ross Upshur, MD[3,4], Sakina Walji, MD[3,37], Rachel Walsh, MD[3,21], Machelle Wilchesky, PhD[47], Sabrina Wong, PhD[48], Brianne Wood, PhD [49, 50], Ryan Zarychanski, MD[5], Barbara Zelek, MD[50], Yoav Keynan, MD[5], Jolanta Piszczek, PharmD[51,52], Daniel Warshafsky, MD[4,53]

List of affiliations:

1. MAP Centre for Urban Health Solutions, Li Ka Shing Knowledge Institute, Unity Health Toronto, Toronto, Ontario, Canada
2. Department of Family and Community Medicine, St. Michael's Hospital, Toronto, Ontario, Canada
3. Department of Family and Community Medicine, Faculty of Medicine, University of Toronto, Toronto, Ontario, Canada
4. Dalla Lana School of Public Health, University of Toronto, Toronto, Ontario, Canada
5. Department of Family Medicine, Max Rady College of Medicine, University of Manitoba, Winnipeg, Manitoba, Canada
6. Nuffield Department of Population Health, University of Oxford, Oxford, England, United Kingdom

7. Memorial University of Newfoundland, St. John's, Newfoundland and Labrador, Canada
8. Department of Medicine, Faculty of Medicine and Health Sciences, McGill University, Montréal, Québec, Canada
9. Department of Family Medicine, University of Calgary, Calgary, Alberta, Canada,
10. Department of Community Health Sciences, University of Calgary, Calgary, Alberta, Canada
11. Faculty of Medicine, University of British Columbia, Vancouver, British Columbia, Canada
12. Centre for Addiction and Mental Health, Toronto, Ontario, Canada
13. Department of Statistics and Actuarial Science, Simon Fraser University, Burnaby, British Columbia, Canada
14. Department of Medicine, Dalhousie University, Halifax, Nova Scotia, Canada
15. Department of Family Medicine, Queen's University, Kingston, Ontario, Canada
16. Department of Community Medicine, Université de Sherbrooke, Sherbrooke, Québec, Canada
17. Nuffield Department of Primary Care Health Sciences, University of Oxford, Oxford, England, United Kingdom
18. University Health Network, Toronto, Ontario, Canada
19. Bruyere Research Institute, Ottawa, Ontario, Canada
20 Department of Family Medicine, University of Ottawa, Ottawa, Ontario, Canada
21. Credit Valley Family Health Team, Mississauga, Ontario, Canada
22. Sunnybrook Health Sciences Centre, University of Toronto, Toronto, Ontario, Canada
23. Department of Family Medicine and Emergency Medicine, Université de Sherbrooke, Sherbrooke, Québec, Canada
24. College of Pharmacy and Department of Community Health and Epidemiology, Dalhousie University, Halifax, Nova Scotia, Canada
25. Women's College Hospital, Toronto, Ontario, Canada
26. Department of Nutritional Sciences, Temerty Faculty of Medicine, University of Toronto, Toronto, Ontario, Canada
27. Division of Endocrinology and Metabolism, St. Michael's Hospital, Toronto, Ontario, Canada
28. Scarborough Health Network, Toronto, Ontario, Canada
29. VITAM Research Center on Sustainable Health, Quebec Integrated University Health and Social Services Center, Québec City, Québec, Canada
30 Faculty of Medicine, Université Laval, Québec City, Québec, Canada
31. Department of Psychiatry, Rady Faculty of Health Sciences, University of Manitoba, Winnipeg, Manitoba, Canada
32. College of Pharmacy, Rady Faculty of Health Sciences, University of Manitoba, Winnipeg, Manitoba, Canada
33. Faculty of Medicine, University of Southampton, Southampton, England, United Kingdom
34. Department of Surgery, Rady Faculty of Health Sciences, University of Manitoba, Winnipeg, Manitoba, Canada
35. Department of Family Medicine and Emergency Medicine, Faculty of Medicine, Université de Montréal, Montréal, Québec, Canada
36. Department of Family Medicine, McMaster University, Hamilton, Ontario, Canada
37. Departments of Surgery and Critical Care Medicine, University of Toronto, Toronto, Ontario, Canada

38. Lawrence Bloomberg Faculty of Nursing, University of Toronto, Toronto, Ontario, Canada
39. Department of Health Research Methods, Evidence, and Impact, McMaster University, Hamilton, Ontario, Canada
40. Department of Academic Family Medicine, University of Saskatchewan, Saskatoon, Saskatchewan, Canada
41. Department of Family Medicine, Schulich School of Medicine and Dentistry, Western University, London, Ontario, Canada
42. Department of Research and Evaluation, Alliance for Healthier Communities, Toronto, Ontario, Canada
43. Department of Respiratory Therapy, Rady Faculty of Health Sciences, University of Manitoba, Winnipeg, Manitoba, Canada
44. Division of Infectious Diseases, University of Alberta, Edmonton, Alberta, Canada
45. Department of Family & Community Medicine, St. Joseph's Health Centre, Toronto, Ontario, Canada
46. Primary Care and Population Health Systems, Humber River Hospital, North York, Ontario, Canada
47. Department of Family Medicine and Division of Geriatric Medicine, McGill University, Montréal, Québec, Canada
48 Centre for Health Services and Policy Research and School of Nursing, University of British Columbia, Vancouver, British Columbia, Canada
49 Thunder Bay Regional Health Research Institute, Thunder Bay, Ontario, Canada
50. Section of Family Medicine, Northern Ontario School of Medicine University, Thunder Bay, Ontario, Canada
51. Department of Pharmaceutical Sciences, University of British Columbia, Vancouver, British Columbia, Canada
52. BC COVID Therapeutics Committee, Vancouver, British Columbia, Canada
53. Office of the Chief Medical Officer of Health, Public Health, Ontario Ministry of Health, Toronto, Ontario, Canada

Principal investigators: Andrew Pinto, Benita (Banafshe) Hosseini, Amanda Condon, Bruno da Costa, Peter Daley, Michelle Greiver, Peter Jüni, Todd C. Lee, Kerry McBrien, Emily McDonald, Srinivas Murthy, Peter Selby
Methods and Statistical Analysis Committee: Bruno da Costa, Peter Jüni (Co-Chair), Clement Ma, John Marshall, Rahim Moineddin, Nav Persaud, Jay Park, Haolun Shi (Co-Chair), and Kevin Thorpe
Trial Steering Committee: Amanda Condon, Bruno da Costa, Peter Daley, Michelle Greiver, Benita Hosseini, Peter Jüni, Todd C. Lee, Emily McDonald, Kerry McBrien, Srinivas Murthy, Andrew Pinto (Chair), and Peter Selby
Data Safety and Monitoring Committee: Nick Daneman, Shirin Golchi, Hensley Mariathas (Chair), Michaeline McGuinty, and Kevin Schwartz
Canadian COVID-19 Therapeutics Advisory Panel: Lisa Barrett, Peter Daley, Mathew Grandy, Jennifer Isenor, Yoav Keynan, Andrew Morris, Jolanta Piszcze (Chair), Fahad Razak, Lynora Saxinger, and Jeoffrey Tranmer

CanTreatCOVID Coordinating Office:

Co-study Leads: Andrew Pinto (ON), Benita Hosseini (ON), Srinivas Murthy (BC), Kerry McBrien (AB), Emily McDonald (QB), Peter Daley (NFLD), Amanda Condon (MB)
Trial Managements: Kawsika Sivayoganathan (ON), Leslie Love/Salima Jutha (BC), Anita Oomen (AB), Kristen Moran (QB), Sherin Paraplammoottil Cheriyan (MB), Valerie Whittle (NFLD)

Clinical Team: Andrew Pinto (ON site investigator & QI), Srinivas Murthy (BC site investigator & QI), Kerry McBrien (AB site investigator & QI), Emily McDonald (QB site investigator & QI), Peter Daley (NFLD site investigator), Amanda Condon (MB site investigator and QI), Peter Selby (study medical monitor)
Database Centralized Support: Applied Health Research Centre (AHRC). Gurpreet Lakhanpal, Rekha Thomas, Maria Naydenova, Marzena Boczulak, Myriam Lafreniere-Roula, Kevin Thorpe
Study monitoring: Christina Sathasivam, Rasha Mahmoud
Statistics: Haolun Shi (Lead), Myriam Lafreniere-Roula, Kevin Thorpe; Postgraduate Trainees: Yan Yichen, Han Yueyang, Jia Shifan, Feng Jiahui
Patient and Community Engagement (CE) team: Brenda Andreas, Cris Carter, Jane Cooney, Jaydee Cossar (BC community engagement specialist), Gabriela Covaci, Amy Ferris (AB community engagement specialist), Letlotlo Gariba, Jennifer Hulme, Veronika Kiryanova, Kathy Kobow, Gillian Kranias (ON community engagement specialist), Mike Lapenna, Mary Liu, Chris Maddison, Dorothy Nelson, Moon Ja Park, Lyric Paul, Donna Rubenstein, Dorothy Senior, Allard Schipper, Kimberly Strain, Margo Twohig, Mike Warren, John Zhan, Alexander Zsager

Participating provinces included: Unity Health Toronto Research Ethics Board – St. Michael's Hospital (Ontario); McGill University Health Centre Research Ethics Board (Quebec); The Conjoint Health Research Ethics Board, University of Calgary (Alberta); The University of Manitoba Biomedical Research Board - Research Ethics Bannatyne (Manitoba); University British Columbia Children's & Women's Research Ethics Board (British Columbia); Newfoundland and Labrador Health Research Ethics Board (Newfoundland and Labrador)

Listing of comorbidities for inclusion

PANORAMIC

- chronic respiratory disease (including chronic obstructive pulmonary disease (COPD), cystic fibrosis and asthma requiring at least daily use of preventative and/or reliever medication);
- chronic heart or vascular disease;
- chronic kidney disease;
- chronic liver disease;
- chronic neurological disease (including dementia, stroke, epilepsy);
- severe and profound learning disability;
- Down’s syndrome;
- diabetes mellitus (Type or Type II);
- immunosuppression: primary (e.g., inherited immune disorders resulting from genetic mutations, usually present at birth and diagnosed in childhood) or secondary due to disease or treatment (e.g., sickle cell, HIV, cancer, chemotherapy);
- solid organ, bone marrow and stem cell transplant recipients;
- morbid obesity (BMI >35);
- severe mental illness;
- care home resident;
- judged by recruiting medically qualified professional, research nurse, nurse prescriber, prescribing pharmacist, dependent on the ISA for the specific IMP involved, to be clinically vulnerable

CanTreatCOVID

- chronic respiratory disease (including COPD, cystic fibrosis and asthma requiring at least daily use of preventative and/or reliever medication);
- chronic heart or vascular disease;
- chronic kidney disease; chronic liver disease;
- chronic neurological disease (including dementia, stroke, epilepsy);
- severe and profound learning disability;
- Down’s syndrome;
- diabetes (Type 1 or Type 2);
- immunosuppression: primary (e.g. inherited immune disorders resulting from genetic mutations) or secondary due to disease or treatment (e.g. sickle cell, HIV, cancer, chemotherapy);
- solid organ, bone marrow and stem cell transplant recipients;
- morbid obesity (BMI >35);
- severe mental illness;
- care home resident

Virology sub study

Those who took part were couriered a kit with nasopharyngeal swabs and blood spot testing kits. Written and video (https://youtu.be/klB-ckiQGz8) instructions for self-sampling were provided. They were asked to post the samples to the virology-processing site (postage and packaging were pre-paid). Participants in the intensive sampling cohort were asked to provide daily nasal or pharyngeal swabs for the first 7 days and on day 14 (or day 13 or 15). In the non-intensive sampling cohort, participants were asked to provide nasal or pharyngeal swabs on days 1, 5 (or day 4 or 6) and 14 (or day 13 or 15). Participants in the nirmatrelvir–ritonavir plus usual care group were asked to take their first sample before the first dose of nirmatrelvir–ritonavir, whereas those in the usual care group were asked to provide their first sample the day after randomisation. All participants in the virology sub study were asked to provide three finger-prick dried blood spot samples, one each on days 1, 5 (or day 4 or 6), and 14 (or day 13 or 15).

Sample size determination

The sample size for the virology sub study was based on simulations from a viral dynamic model from early 2020,[10] which suggested that inclusion of 30 patients per group would detect a 2·5 times increase in viral clearance (which translates into roughly double the rate of undetectable viral loads at day 7) in patients who started treatment within 5 days of symptom onset (with 90% power and an α of 0·05) compared with those receiving usual care. Clinical improvement could be associated with smaller decreases in viral load, and viral dynamic modelling leveraging time-series viral-load data can detect much smaller drug effect sizes.[11]

Adverse event reporting

Standard adverse event data were not routinely captured in the PANORAMIC trial. Our strategy was to comprehensively capture safety data for serious adverse events and adverse events for which data are scarce. There was, however, a robust mechanism in place for participants to seek advice on the management of troublesome adverse events, with 24-hour clinical advice about trial participation and medication available to participants. In the PANORAMIC -trial all non-COVID events (at the discretion of the reporting nurse clinician) in the nirmatrelvir–ritonavir arm reported in the safety or follow-up calls or the daily diary were reported as adverse events. In the CanTreatCOVID trial any clinical event or worsening of a pre-existing condition were reported to site investigators and recorded as adverse events.

Sample size reconsiderations for the PANORAMIC trial

The original sample size was estimated to be 5300 participants per arm. The sample size provides approximately 90% power for detecting a 33% relative reduction in hospitalization/death in an experimental arm relative to Usual Care. This assumes of an underlying 3% combined hospitalization/death rate in the Usual Care arm, and an intervention lowering this to 2%, with some adjustments for the multiple interim analyses. As the trial progress, the total sample size has been adjusted to account for a lower than expected overall event rate. Table 1 presented a range of sample sizes based on different event rate in Usual Care with the same estimated effect size, as specified in the protocol. Should the event rate be lower than expected then the sample size will increase with advice from the Trial Steering Committee.

Table S1 Power and sample size estimates for PANORAMIC per treatment arm based on 33% relative reduction

90% power			80% power		
**Usual Care**	**Treatment**	**Sample size**	**Usual Care**	**Treatment**	**Sample size**
1.0%	0.67%	16578	1.0%	0.67%	12534
1.5%	1.0%	10771	1.5%	1.0%	8145
2.0%	1.3%	7241	2.0%	1.3%	5480
3.0%	2.0%	5319	3.0%	2.0%	4023
4.0%	2.7%	4177	4.0%	2.7%	3159
5.0%	3.4%	3425	5.0%	3.4%	2590

Due to decreasing in hospitalization/death rate, the following table provides different sample size options based on an overall event rate of 0.8% with varying base rate:

Event rate in UC (R1)	Event rate in Treatment group (R2)	Overall event rate	Relative risk (R1/R2)	90% (per arm)	80% (per arm)
1%	0.6%	0.8%	0.60	10916	8278
1.1%	0.5%	0.8%	0.45	4959	3786
1.2%	0.4%	0.8%	0.33	2849	2189
1.3%	0.3%	0.8%	0.23	1861	1438

Recruitment overlaps in PANORAMIC

There was an overlap of 7 days between the period where the Molnupiravir arm was open and during that time 18 participants were recruited who were eligible to both Molnupiravir and Nirmatrelvir-ritonavir, of whom 5 were randomized to usual care and 9 to Nirmatrelvir-ritonavir. Given the small amount of overlap we exclude usual care participants randomized during the time that Nirmatrelvir-ritonavir was open but who were only eligible to Molnupiravir.

Early termination of recruitment in CanTreatCOVID

Recruitment was stopped on 30 Sept 2024. The decision was made by the Trial Steering Committee after discussion with the Data Safety and Monitoring Committee, based on likely power, feasibility and available time and resources. At the start of the trial, CanTreatCOVID was supported by a fully funded supply of nirmatrelvir–ritonavir provided through the Public Health Agency of Canada (PHAC). However, this supply was discontinued as of May 31, 2024, when PHAC ceased purchasing nirmatrelvir–ritonavir for use in Canada. In addition, there was a decline in access to testing for SARS-CoV-2 and declining enrollment.

Reasons for not eligible at screening stage

PANORAMIC

Table S2 Reasons for not eligible from patients online for eligibility (n=51,042) (can be more than one reasons) [PANORAMIC]

Reasons (can by more than one)	n
No symptoms of COVID-19	18,136
Not had a PCR test positive for COVID	14,982
Age 18-49 but no comorbidity	13,932
Did not complete all online screening criteria to assess eligibility	4,893
Not willing to use contraception for 28 days	3,134
Symptoms beyond 5 days	2,987
PCR more than 2 days before symptoms	891
Taking part in a PANORAMIC	674
Not willing to take a pregnancy test prior to starting trial medication	642
Unable to give consent	534
Taking part in a RCT of meds for this episode of COVID-19 illness	385
Unable to have legal representative to give consent	373
Paxlovid only: CKD stage 2, 3,4,or 5	324
Age < 18 years	269
Pregnant or planning on becoming pregnant within the next few weeks	264
Breastfeeding or planning on starting during the course of the trial	255
Currently taking Paxlovid	206
Paxlovid only: CKD 2 but did not have eGFR measurement in the past 6 months	165
Known allergy to Molnupiravir	112
Currently admitted to hospital	111
Currently taking Molnupiravir	79
Paxlovid only: Severe liver impairment such as ascites	52
Paxlovid only: Current known rare hereditary problem of galactose intolerance	38
Known allergy to Paxlovid	27

Table S3 Reasons for not eligible assessed by GP (n=42,137) [PANORAMIC]

Reason	n
Symptoms assessed at interview as present for >5 days	38,946
Drug interaction	1,662
Not on highly effective contraceptives	364
Insufficient details to check eligibility	257
No listed preexisting condition	233
No SCR	183
Not eligible (No reason provided)	107
Duplicate	101
Not eligible (no reason recorded)	65
Asymptomatic	51
Lack capacity	23
Declined access to SCR	20
Not COVID positive/no results	20
Mild symptoms	19
Doesn't speak English	18
Pregnant / planning to get pregnant	18
Already on another trial	16
Admitted to hospital	15
Currently breastfeeding	5
Previous participant	4
PCR/LFT results outside window	3
Declined access to SCR/ Not on SCR	2
Not eligible by GP	2
Requires hospital admission	2
Current taking part in other trial	1

Table S4 List of medication that patients were not eligible due to interaction with nirmatrelvir–ritonavir (Can have more than one medications) [PANORAMIC]

Medication	N
Atorvastatin	563
Amlodipine	151
Simvastatin	90
Anticoagulant	65
Salmeterol	58
Apixaban	54
Diazepam	49
Tamsulosin	46
Ivabradine	37
Lercanidipine	36
Quetiapine	35
Rivaroxaban	35
Amitriptyline	33
Sildenafil	30
Other calcium channel blocker	27
Sulfasalazine	21
Tadalafil	18
Fexofenadine	18
Fentanyl	15
Mirtazapine	14
Methylphenidate	13
Fluoxetine	13
Carbamazepine	12
Flecainide	11
Triazodone	11
Tacrolimus	10
Rosuvastatin	9
Clonazepam	9
Dexamfetamine	9
Solifenacin	9
Oxycodone	8
Verapamil	8
Statin	6
Erythromycin	6
Ticagrelor	6
Ranolazine	5
Tolterodine	5
Cyclosporine	5
Aripiprazole	5
Alfuzosin	4
Digoxin	4
Phenytoin	4
Amiodarone	3
Colchicine	3
Eletriptan	3
Flibanserin	3
Imipramine	3
Itraconazole	3
Dabigatran	2
Eplerenone	2
Midazolam	2
St John's wort	2
Vardenafil	2
Abemaciclib	1
Primidone	1
Rifampicin	1
Tofacitinib	1
Estradiol	1
Fesoterodine	1
Did not specify	144

Table S5 Reasons for not eligible from patients self-assessed online - can have multiple reasons (n=21,100) [CanTreatCOVID]

Reasons	n
Intake form not completed	12,060
No covid-19 symptoms beginning in the last 5 days	10,198
Did not have a positive PCR-test or Rapid Antigen Test (RAT)	5,854
Age less than 50 but no chronic conditions	2,296

Table S6 Reasons for not eligible from study team assessment (n=1,276) [CanTreatCOVID]

Reasons	n
Unable to contact participants/screening not initiated	20
Aged 18-49 with no chronic conditions	169
Symptoms onset occurred more than five days prior or did not have a positive SARS-CoV2 test	660
Already received/receiving Paxlovid for COVID	82
Medical reasons	56
Contraindication to study drug (based on assessment by study pharmacist & QI)	51
No longer wish to take part	43
Other reasons	195

Table S7 List of medication that patients were not eligible due to interaction with Paxlovid (can have more than one medications) [CanTreatCOVID]

Medication	N
Clonazepam	5
Aripiprazole	5
Apixaban	4
Rivaroxaban	3
Dextroamphetamine	3
Salmeterol	2
Quetiapine	2
Diltiazem	2
Trazodone	2
Atorvastatin	1
Rosuvastatin	1
Solifenacin	1
Oxycodone	1

Medication	N
Amiodarone	1
Domperidone	1
Lamborexant	1
Brexipiprazole	1
Clarithromycin	1
Edoxaban	1
Atogepant	1
Lisdexamfetamine	1
Zopiclone	1
Buspirone	1
Nifedipine	1
Ubrogepant	1

Representativeness of Study Populations

Table S8 Summary of generalizability of findings for PANORAMIC and CanTreatCOVID

**Disease under investigator**	SARS-CoV-2 infection
**Special considerations related to**	
Sex and gender	Despite similar incidence between males and females, adult males have been found to be at higher risk of progressing to severe COVID-19, including hospitalization, ICU admission, organ failure, and death, compared to females. Transgender individuals were also found to be higher risk of poor outcomes. Gender identity has been associated with poorer outcomes is some retrospective studies but the availability of data is limited.
Age	Age has been consistently associated with poor outcomes, with the risk of severe illness, hospitalization, intensive care, and death increasing sharply with age, particularly after the age of 60.
Ethnicity or race	Minority ethnic minority groups are at higher risk of worse outcomes, with differences especially marked early on in the pandemic, prior to widespread vaccination.
Social economics	People living in more deprived or disadvantaged areas, including in the UK and Canada, generally at higher risk of hospital admission, severe illness and death.
**Generalizability of findings**	In PANORAMIC, nearly 20% more females than males were randomised. We did not collect information on gender. Over 98% had been vaccinated at least once. The mean age just less that 54 years. About 94% were white, and just over 2% Asian, 2% mixed race and 0.5% Black. Overall in the UK, 17% of those aged 18 and over identify as belonging to a minority ethic group. This proportion reduces with age, with around 14% of those aged 50 to 59, and 7% percent of those aged 60 to 69 from a minority ethnic background. Around 17% were obese and around 65% had a comorbidity. Thus females and those from a minority ethnic background are under represented in the PANORAMIC population.<br><br>In CanTreatCOVID, the majority of participants identified as female (nirmatrelvir-ritonavir arm: 66.2%, usual care arm: 64.5%), and one participant selected "other" as their sex (0.3% in the nirmatrelvir-ritonavir arm). The mean age was just under 55 years. 81% participants in the nirmatrelvir-ritonavir arm and 76% in the usual care arm identified as white, with smaller percentages from Asian (10%-14%), mixed race, Black, and other ethnic categories. Around 55% of participants were employed full time or part time, which is consistent across both arms. 10.6% from the nirmatrelvir-ritonavir group and 12.8% from the control group make less than $40,000 a year, compared to approximately 30% of the Canadian population. Thus, males, those from minority ethnic backgrounds, and those living on low income are unrepresented in the CanTreatCOVID population.

Supplementary Tables and Figures for the PANORAMIC

Definitions of the secondary outcomes reported below and details of analysis can be found in the statistical analysis plan.

Table S9 Symptoms and comorbidities at baseline [PANORAMIC]

	PANORAMIC		CanTreatCOVID	
	**Nirmatrelvir-ritonavir (N=1736)**	**Control (N=1768)**	**Nirmatrelvir-ritonavir (N=358)**	**Control (N=358)**
**Baseline symptoms**				
**Shortness of breath, n(%)**				
No problem	671 (38.7%)	728 (41.2%)	203 (56.7%)	181 (50.6%)
Minor problem	632 (36.4%)	628 (35.5%)	103 (28.8%)	110 (30.7%)
Moderate problem	372 (21.4%)	363 (20.5%)	41 (11.5%)	34 (9.5%)
Major problem	61 (3.5%)	49 (2.8%)	9 (2.5%)	5 (1.4%)
Missing, n(%)	0	0	2 (0.6%)	28 (7.8%)
**Fatigue, n(%)**				
No problem	85 (4.9%)	89 (5.0%)	21 (5.9%)	16 (4.5%)
Minor problem	426 (24.5%)	465 (26.3%)	107 (29.9%)	82 (22.9%)
Moderate problem	782 (45.0%)	802 (45.4%)	156 (43.6%)	141 (39.4%)
Major problem	443 (25.5%)	412 (23.3%)	72 (20.1%)	91 (25.4%)
Missing, n(%)	0	0	2 (0.6%)	28 (7.8%)
**Muscle ache, n(%)**				
No problem	319 (18.4%)	359 (20.3%)	75 (20.9%)	71 (19.8%)
Minor problem	571 (32.9%)	549 (31.1%)	146 (40.8%)	120 (33.5%)
Moderate problem	601 (34.6%)	610 (34.5%)	103 (28.8%)	112 (31.3%)
Major problem	245 (14.1%)	250 (14.1%)	32 (8.9%)	27 (7.5%)
Missing, n(%)	0	0	2 (0.6%)	28 (7.8%)
**Vomiting, n(%)**				
No problem	1250 (72.0%)	1292 (73.1%)	259 (72.3%)	255 (71.2%)
Minor problem	345 (19.9%)	344 (19.5%)	79 (22.1%)	53 (14.8%)
Moderate problem	126 (7.3%)	120 (6.8%)	15 (4.2%)	17 (4.7%)
Major problem	15 (0.9%)	12 (0.7%)	3 (0.8%)	5 (1.4%)
Missing, n(%)	0	0	2 (0.6%)	28 (7.8%)
**Diarrhoea, n(%)**				
No problem	1341 (77.2%)	1373 (77.7%)	-	-
Minor problem	268 (15.4%)	270 (15.3%)	-	-

	PANORAMIC		CanTreatCOVID	
	**Nirmatrelvir-ritonavir (N=1736)**	**Control (N=1768)**	**Nirmatrelvir-ritonavir (N=358)**	**Control (N=358)**
Moderate problem	107 (6.2%)	96 (5.4%)	-	-
Major problem	20 (1.2%)	29 (1.6%)	-	-
**Loss of smell or taste, n(%)**				
No problem	1004 (57.8%)	995 (56.3%)	242 (67.6%)	203 (56.7%)
Minor problem	395 (22.8%)	440 (24.9%)	68 (19%)	83 (23.2%)
Moderate problem	197 (11.3%)	196 (11.1%)	29 (8.1%)	27 (7.5%)
Major problem	140 (8.1%)	137 (7.7%)	17 (4.7%)	17 (4.7%)
Missing, n(%)	0	0	2 (0.6%)	28 (7.8%)
**Headache, n(%)**				
No problem	297 (17.1%)	342 (19.3%)	-	-
Minor problem	595 (34.3%)	625 (35.4%)	-	-
Moderate problem	605 (34.9%)	557 (31.5%)	-	-
Major problem	239 (13.8%)	244 (13.8%)	-	-
**Dizziness, n(%)**				
No problem	951 (54.8%)	978 (55.3%)	-	-
Minor problem	478 (27.5%)	496 (28.1%)	-	-
Moderate problem	236 (13.6%)	237 (13.4%)	-	-
Major problem	71 (4.1%)	57 (3.2%)	-	-
**Abdominal pain, n(%)**				
No problem	1330 (76.6%)	1334 (75.5%)	-	-
Minor problem	277 (16.0%)	311 (17.6%)	-	-
Moderate problem	116 (6.7%)	109 (6.2%)	-	-
Major problem	13 (0.7%)	14 (0.8%)	-	-
**Generally unwell, n(%)**				
No problem	26 (1.5%)	39 (2.2%)	-	-
Minor problem	448 (25.8%)	438 (24.8%)	-	-
Moderate problem	883 (50.9%)	911 (51.5%)	-	-
Major problem	379 (21.8%)	380 (21.5%)	-	-
Missing, n(%)	0	0	-	-
**Fever, n(%)**				
No problem	528 (30.4%)	560 (31.7%)	158 (44.1%)	140 (39.1%)
Minor problem	682 (39.3%)	706 (39.9%)	126 (35.2%)	119 (33.2%)
Moderate problem	439 (25.3%)	409 (23.1%)	62 (17.3%)	62 (17.3%)

	PANORAMIC		CanTreatCOVID	
	Nirmatrelvir-ritonavir (N=1736)	Control (N=1768)	Nirmatrelvir-ritonavir (N=358)	Control (N=358)
Major problem	87 (5.0%)	93 (5.3%)	10 (2.8%)	9 (2.5%)
Missing, n(%)	0	0	2 (0.6%)	28 (7.8%)
**Cough, n(%)**				
No problem	182 (10.5%)	182 (10.3%)	44 (12.3%)	36 (10.1%)
Minor problem	757 (43.6%)	789 (44.6%)	162 (45.3%)	136 (38%)
Moderate problem	658 (37.9%)	649 (36.7%)	120 (33.5%)	124 (34.6%)
Major problem	139 (8.0%)	148 (8.4%)	30 (8.4%)	34 (9.5%)
Missing, n(%)	-	-	2 (0.6%)	28 (7.8%)
**Comorbidities**				
**Lung disease, n(%)**	454 (26.2%)	439 (24.8%)	70 (19.6%)	58 (16.2%)
Missing, n(%)	0 (0.0%)	0 (0.0%)	2 (0.6%)	27 (7.5%)
**Heart disease, n(%)**	83 (4.8%)	63 (3.6%)	6 (1.7%)	6 (1.7%)
Missing, n(%)	0 (0.0%)	0 (0.0%)	2 (0.6%)	27 (7.5%)
**Kidney disease, n(%)**	9 (0.5%)	7 (0.4%)	1 (0.3%)	2 (0.6%)
Missing, n(%)	0 (0.0%)	0 (0.0%)	2 (0.6%)	27 (7.5%)
**Liver disease, n(%)**	17 (1.0%)	17 (1.0%)	4 (1.1%)	5 (1.4%)
Missing, n(%)	0 (0.0%)	0 (0.0%)	2 (0.6%)	27 (7.5%)
**Neurological disease, n(%)**	60 (3.5%)	66 (3.7%)	6 (1.7%)	5 (1.4%)
Missing, n(%)	0 (0.0%)	0 (0.0%)	2 (0.6%)	27 (7.5%)
**Learning disability, n(%)**	8 (0.5%)	8 (0.5%)	-	-
**Down's syndrome, n(%)**	6 (0.3%)	5 (0.3%)	-	-
**Diabetes, n(%)**	132 (7.6%)	150 (8.5%)	86 (24.0%)	74 (20.7%)
Missing, n(%)	0 (0.0%)	0 (0.0%)	2 (0.6%)	27 (7.5%)
**Weakened immune system‡, n(%)**	152 (8.8%)	166 (9.4%)	9 (2.5%)	15 (4.2%)
Missing, n(%)	0 (0.0%)	0 (0.0%)	2 (0.6%)	27 (7.5%)
**Transplant recipient, n(%)**	11 (0.6%)	7 (0.4%)	0 (0.0%)	0 (0.0%)
**Obesity, n(%)**	293 (16.9%)	312 (17.6%)	58 (16.2%)	56 (15.6%)
Missing, n(%)	0 (0.0%)	0 (0.0%)	2 (0.6%)	27 (7.5%)
**Mental illness, n(%)**	32 (1.8%)	25 (1.4%)	-	-
**Hypertension, n(%)**	221 (12.7%)	253 (14.3%)	56 (15.6%)	68 (19.0%)
Missing, n(%)	0 (0.0%)	0 (0.0%)	2 (0.6%)	27 (7.5%)
**Other vulnerability, n(%)**	329 (19.0%)	349 (19.7%)	-	-

Table S10 Secondary outcomes [PANORAMIC]

Outcome	Nirmatrelvir-ritonavir	Usual Care	Estimated treatment effect (95% BCI)
**Time to sustained recovery**			
Number recovered by day 28, n/N(%)	1044/1690 (61.8%)	821/1646 (49.9%)	
Median time to recovery, days, median (IQR)	21 (10 to not reached)*	27 (15 to not reached) *	
Hazard ratio (95% credible interval) for each time interval using a time varying piecewise exponential model			
Time interval 1 to 2 days			0.98 (0.38 to 2.44)†
3 to 7 days			2.02 (1.62 to 2.52)†
8 to 11 days			1.57 (1.28 to 1.96)†
12 to 28 days			1.20 (1.07 to 1.35)†
**Time to alleviation of all symptoms**			
Number alleviated by day 28, n/N(%)	1371/1496 (91.6%)	1257/1442 (87.2%)	
Median time to alleviation, days, median (IQR)	4 (3 to 8)*	5 (3 to 11)*	
Hazard ratio (95% credible interval) for each time interval using a time varying piecewise exponential model			
Time interval 1 to 2 days			1.13 (0.97 to 1.32)†
3 to 7 days			1.46 (1.31 to 1.64)†
8 to 11 days			1.22 (0.98 to 1.51)†
12 to 28 days			0.88 (0.70 to 1.10)†
**Time to sustained alleviation of all symptoms**			
Number alleviated by day 28, n/N(%)	1271/1496 (85.0%)	1143/1442 (79.3%)	
Median time to alleviation, days, median (IQR)	8 (3 to 21)*	12 (5 to 25)*	
Hazard ratio (95% credible interval) for each time interval using a time varying piecewise exponential model			
Time interval 1 to 2 days			1.33 (1.08 to 1.64)†
3 to 7 days			1.56 (1.36 to 1.79)†
8 to 11 days			0.92 (0.75 to 1.14)†
12 to 28 days			1.08 (0.94 to 1.24)†

Outcome	Nirmatrelvir-ritonavir	Usual Care	Estimated treatment effect (95% BCI)
**Time to Initial reduction of severity of symptoms**			
Number alleviated by day 28, n/N(%)	1415/1657 (85.4%)	1202/1599 (75.2%)	
Median time to alleviation, days, median (IQR)	8 (5 to 14)*	12 (7 to 25)*	
Hazard ratio (95% credible interval) for each time interval using a time varying piecewise exponential model			
Time interval 1 to 2 days			1.25 (0.88 to 1.79)†
3 to 7 days			1.70 (1.51 to 1.92)†
8 to 11 days			1.73 (1.47 to 2.03)†
12 to 28 days			1.11 (0.97 to 1.28)†
Rating of how well participant feels (0 worst, 10 best), mean (SD)[n]			
Day 7	7.0 (1.8) [1590]	6.5 (1.8) [1490]	0.53 (0.41 to 0.64)‖
Day 14	7.7 (1.8) [1543]	7.3 (1.8) [1445]	0.39 (0.274to 0.51)‖
Day 21	8.0 (1.7) [1454]	7.8 (1.7) [1354]	0.22 (0.10 to 0.34)‖
Day 28	8.2 (1.6) [1504]	8.0 (1.7) [1420]	0.24 (0.12 to 0.35)‖
New infections in household, n/N (%)	427/1484 (28.8%)	444/1434 (31.0%)	0.91 (0.77 to 1.06) ¶
Number of new infections over 28 days, median (IQR)	0 (0 to 1) **	0 (0 to 1) **	0.94 (0.84 to 1.06) ††
Virology- Intensive samples cohort			
Viral load below detection level, n/N (%) Day 2	2/31 (6.5)	0/33 (0.0)	
Day 3	3/32 (9.4)	2/33 (6.1)	
Day 4	4/32 (12.5)	2/33 (6.1)	
Day 5	9/32 (28.1)	6/33 (18.2)	2.00 (0.57 to 7.61) ‡‡
Day 6	15/31 (48.4)	8/33 (24.2)	3.58 (1.18 to 11.58)
Day 7	12/32 (37.5)	10/33 (30.3)	1.53 (0.52 to 4.56)
Viral load, geometric mean(geometric SD) Day 2	447815.8 (61.6)	892248.4 (32.7)	0.47 (0.12 to 1.86) ‖‖
Day 3	115687.1 (57.9)	202542.1 (58.1)	0.478 (0.117 to 1.91) ‖‖
Day 4	19490.9 (25.8)	44420.9 (33.0)	0.35 (0.09 to 1.48) ‖‖
Day 5	2802.0 (13.8)	21080.7 (41.7)	0.11 (0.038 to 0.45) ‖‖
Day 6	898.5 (11.6)	3353.2 (21.4)	0.20 (0.06 to 0.83) ‖‖
Day 7	759.3 (10.8)	3095.2 (30.0)	0.21 (0.05 to 0.79) ‖‖

NB: All credible interval widths for the outcomes have not been adjusted for multiplicity and cannot be used to infer definitive treatment effects.
* Kaplan-Meier estimates of median time to event and interquartile range from the raw data.
†Estimated HR derived from a time varying piecewise exponential model adjusted for age, comorbidity and vaccination status. HR > 1 favours Nirmatrelvir-ritonavir
‡ Estimated HR derived from a piecewise exponential model adjusted for age, comorbidity and vaccination status. HR > 1 favours Nirmatrelvir-ritonavir
|| Bayesian linear mixed effect model adjusted for age, comorbidity and vaccination status. Participant fitted as a random effect. Estimated mean difference > 0 favours nirmatrelvir-ritonavir .
¶Bayesian logistic regression, adjusted for, age, comorbidity and vaccination status
**Median and interquartile range from raw data.
††Bayesian Poisson regression adjusted for age, comorbidity and vaccination status and number of people in household at baseline as an offset
‡‡ Bayesian logistic regression adjusting for sex, age, and baseline log_{10}(viral load). Adjusted OR > 1 favours nirmatrelvir-ritonavir. NOTE: Mixed effects logistic regression model did not converge.
|| || Bayesian mixed effect model for log_{10}(viral load) adjusting for sex, age, and baseline log_{10}(viral load); estimates were transformed to adjusted geometric ratio < 1 favours nirmatrelvir-ritonavir fitted with main effects of treatment, day and a treatment x day interaction in order to estimate time specific treatment effects.

Health and Social Care Service Use

1. Any contact with health care services is defined as reporting use of that service at any point in the daily diary or calls. An odds ratio<1 favours nirmatrelvir-ritonavir
2. Number of contacts with healthcare services is the total count of uses across daily diaries and calls. The median along with the interquartile range is presented for those participants reporting at least one contact. A rate ratio<1 favours nirmatrelvir-ritonavir.

Table S11 Self-reported contacts with healthcare services [PANORAMIC]

Outcome	Nirmatrelvir-ritonavir	Usual Care	Median Estimate (rate ratio), 95% Bayesian credible interval
Any contact with NHS 111	84/1690 (5%)	134/1646 (8.1%)	0.598 (0.445 to 0.781)
Number of contacts with NHS 111	1 (1 to 1) [1 to 3]	1 (1 to 1) [1 to 5]	0.596 (0.464 to 0.755)
Any contact with GP	393/1689 (23.3%)	457/1646 (27.8%)	0.796 (0.677 to 0.932)
Number of contacts with GP	1 (1 to 2) [1 to 14]	1 (1 to 2) [1 to 10]	0.909 (0.821 to 1.002)
Any contact with ambulance service (not hospitalized)	42/1690 (2.5%)	43/1642 (2.6%)	0.978 (0.625 to 1.456)
Number of contacts with ambulance service	1 (1 to 1) [1 to 2]	1 (1 to 1) [1 to 5]	0.807 (0.530 to 1.182)
Any contact with community nurse	36/1690 (2.1%)	38/1646 (2.3%)	0.967 (0.590 to 1.496)
Number of contacts with community nurse	1 (1 to 1) [1 to 11]	1 (1 to 1) [1 to 4]	1.282 (0.852 to 1.855)
Any contact with physiotherapist	15/1690 (0.9%)	17/1646 (1%)	0.908 (0.426 to 1.716)
Number of contacts with physiotherapist	1 (1 to 1) [1 to 5]	1 (1 to 1) [1 to 5]	0.689 (0.368 to 1.166)
Any contact with counsellor	14/1690 (0.8%)	15/1646 (0.9%)	0.942 (0.428 to 1.801)
Number of contacts with counsellor	1 (1 to 3) [1 to 18]	1 (1 to 2) [1 to 6]	1.370 (0.838 to 2.165)
Any contact with social worker	4/1690 (0.2%)	4/1646 (0.2%)	1.118 (0.230 to 3.311)
Number of contacts with social worker	1 (1 to 1) [1 to 1]	1 (1 to 3) [1 to 5]	0.551 (0.131 to 1.448)
Any contact with home carer	10/1690 (0.6%)	8/1646 (0.5%)	1.333 (0.467 to 3.000)

Outcome	Nirmatrelvir-ritonavir	Usual Care	Median Estimate (rate ratio), 95% Bayesian credible interval
Number of contacts with home carer	3 (1 to 4) [1 to 28]	3 (1 to 18) [1 to 27]	0.877 (0.619 to 1.221)
Any contact with occupational therapist	31/1690 (1.8%)	36/1646 (2.2%)	0.858 (0.512 to 1.351)
Number of contacts with occupational therapist	1 (1 to 1) [1 to 1]	1 (1 to 1) [1 to 2]	0.737 (0.452 to 1.135)
Any contact with hospital A&E	99/1690 (5.9%)	87/1646 (5.3%)	1.142 (0.837 to 1.531)
Number of contacts with hospital A&E	1 (1 to 1) [1 to 7]	1 (1 to 1) [1 to 3]	1.123 (0.863 to 1.445)
Any contact with respiratory outpatient clinic	28/1689 (1.7%)	35/1646 (2.1%)	0.812 (0.474 to 1.303)
Number of contacts with respiratory outpatient clinic	1 (1 to 1) [1 to 2]	1 (1 to 1) [1 to 5]	0.717 (0.444 to 1.102)
Any contact with hospital at home for COVID-19	40/1690 (2.4%)	37/1646 (2.2%)	1.109 (0.689 to 1.696)
Number of contacts with hospital at home for COVID-19	1 (1 to 1) [1 to 6]	1 (1 to 3) [1 to 28]	0.382 (0.277 to 0.507)
Any contact with other services	58/1690 (3.4%)	96/1646 (5.8%)	0.590 (0.414 to 0.815)
Number of contacts with other services	1 (1 to 3) [1 to 7]	1 (1 to 2) [1 to 11]	0.716 (0.557 to 0.904)

NB: All credible interval widths for the outcomes have not been adjusted for multiplicity and cannot be used to infer definitive treatment effects.

*Bayesian logistic regression, adjusted for, age, comorbidity and vaccination status

†Bayesian Poission regression, adjusted for, age, comorbidity and vaccination status and using number of people living with the participant at baseline as the offset.

Kaplan-Meier Plots for time to event outcomes. Proportional hazards assumptions for time to event outcomes were assessed using a test of the Schoenfeld residuals and visual inspection of the log-log plots. For time to recovery and time to sustained recovery the proportional hazards assumptions were found to have been violated.

Figure S1 Kaplan-Meier curves for time to recovery [PANORAMIC]

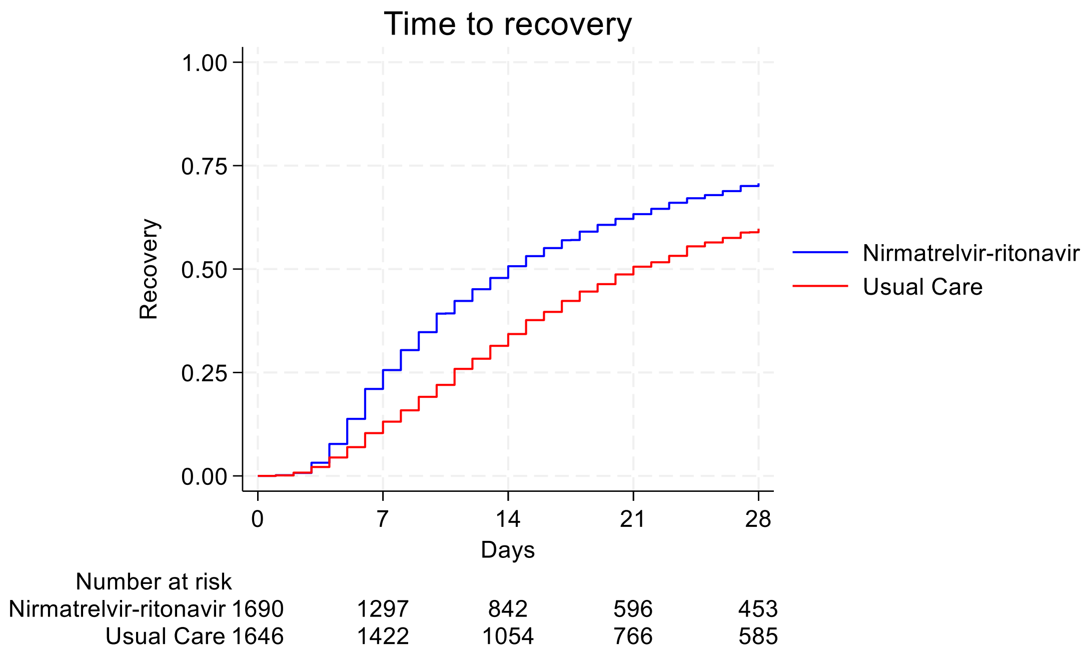

Figure S2 Kaplan-Meier curves for time to sustained recovery [PANORAMIC]

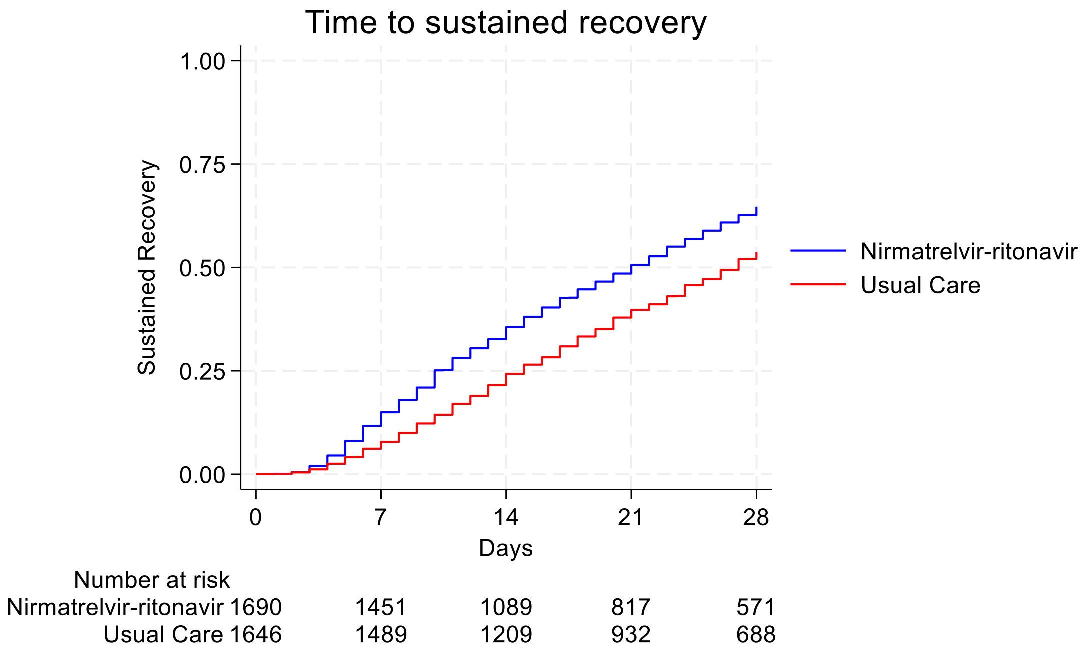

Analysis of Individual Symptoms

The analysis of the PANORAMIC dataset includes all data up to day 28 but for the combined analysis outcomes are censored at day 14 to allow comparability of the data with the CanTreatCOVID trial. NB: All credible interval widths presented in the graphs have not been adjusted for multiplicity and cannot be used to infer definitive treatment effects.

Figure S3 Time to alleviation of symptoms [PANORAMIC]

Symptoms	Nirmatrelvir-ritonavir*	Usual Care*	Adjusted HR (95% BCI)
Fever	511/513	444/448	1.25 (1.10, 1.42)
Cough	763/776	689/716	1.33 (1.20, 1.47)
Shortness of breath	401/419	339/356	1.23 (1.07, 1.41)
Fatigue	1115/1191	1001/1111	1.23 (1.13, 1.34)
Muscle ache	796/824	747/776	1.21 (1.10, 1.33)
Nausea and vomiting	131/134	112/114	1.14 (0.90, 1.44)
Diarrhoea	123/124	108/110	1.10 (0.87, 1.40)
Loss of smell or taste	306/329	269/294	1.10 (0.93, 1.28)
Headache	808/819	710/727	1.18 (1.06, 1.30)
Dizziness	289/298	248/260	1.22 (1.04, 1.44)
Abdominal pain	121/126	98/102	1.05 (0.82, 1.34)
Generally unwell	1188/1228	1134/1181	1.17 (1.02, 1.35)
All baseline symptoms	1371/1496	1257/1442	1.25 (1.16, 1.34)

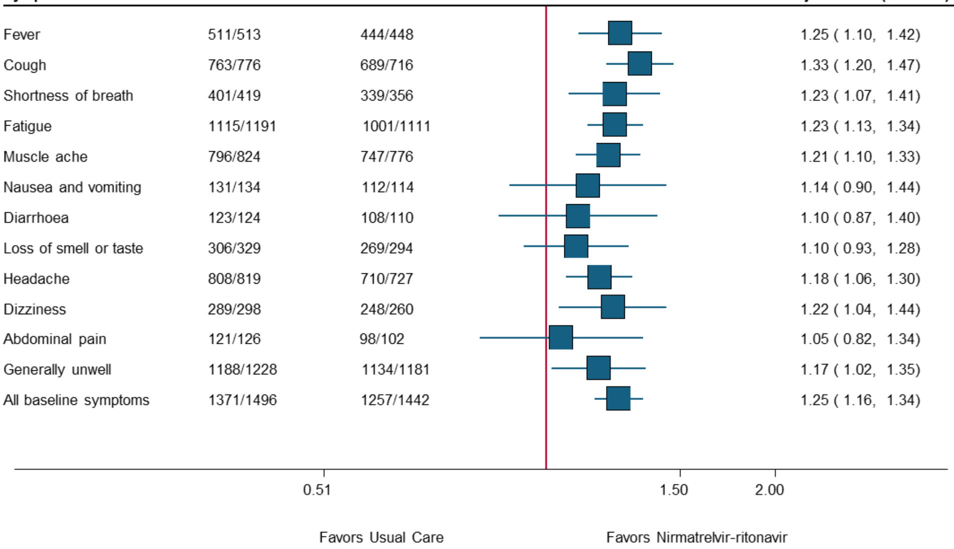

*** Number of alleviation of symptoms by day 28/total reported severe at baseline**

Figure S4 Time to sustained alleviation of symptoms [PANORAMIC]

Symptoms	Nirmatrelvir-ritonavir*	Usual Care*	Adjusted HR (95% BCI)
Fever	510/513	436/448	1.29 (1.14, 1.46)
Cough	736/776	661/716	1.31 (1.18, 1.46)
Shortness of breath	377/419	308/356	1.40 (1.21, 1.62)
Fatigue	1048/1191	916/1111	1.26 (1.15, 1.39)
Muscle ache	764/824	707/776	1.26 (1.14, 1.40)
Nausea and vomiting	129/134	110/114	1.12 (0.88, 1.41)
Diarrhoea	119/124	106/110	1.00 (0.78, 1.28)
Loss of smell or taste	295/329	251/294	1.15 (0.98, 1.35)
Headache	785/819	689/727	1.20 (1.08, 1.33)
Dizziness	280/298	236/260	1.12 (0.95, 1.32)
Abdominal pain	117/126	96/102	1.02 (0.79, 1.32)
Generally unwell	1135/1228	1073/1181	1.20 (1.10, 1.30)
All baseline symptoms	1271/1496	1143/1442	1.23 (1.13, 1.33)

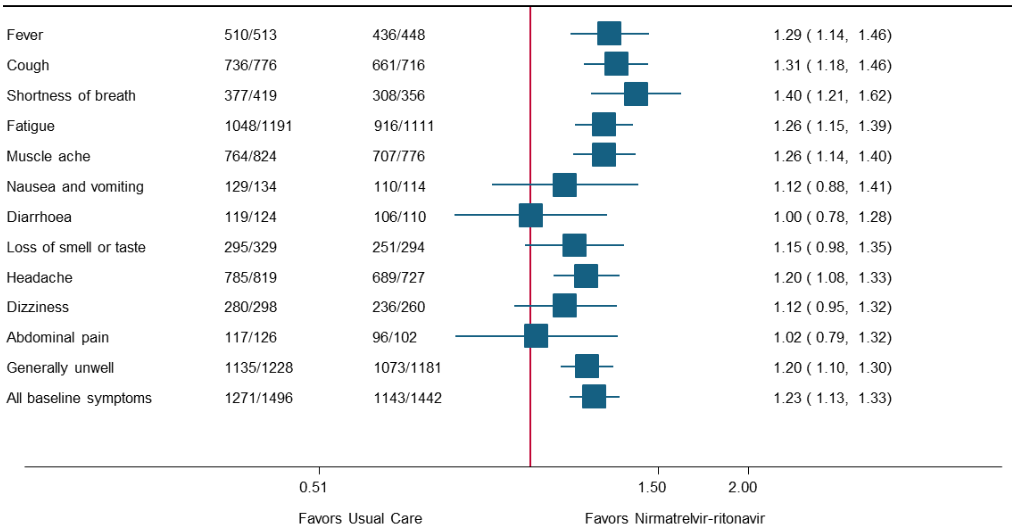

*** Number of sustained alleviation of symptoms by day 28/total reported severe at baseline**

NB: All credible interval widths presented in the graphs have not been adjusted for multiplicity and cannot be used to infer definitive treatment effects.

Figure S5 Time to reduction of symptoms [PANORAMIC]

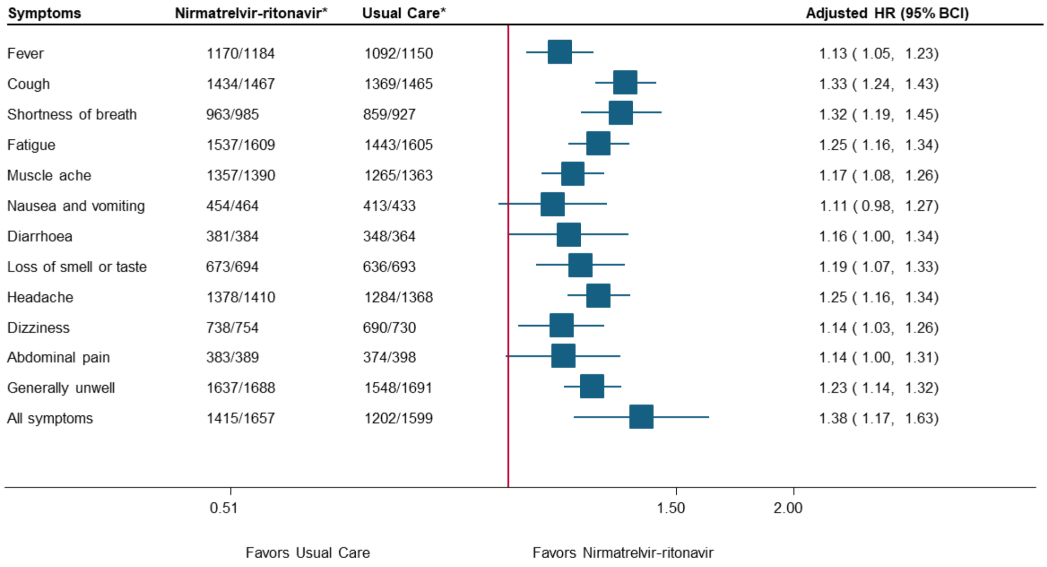

*** Number of initial reduction of symptom by day 28/total reported at least mild symptom at baseline**

Figure S6 Recurrence of any symptom [PANORAMIC]

Effects mostly favour nirmatrelvir-ritonavir, except for nausea and vomiting, diarrhoea and loss of smell or taste. These three symptoms are possible side effects of nirmatrelvir-ritonavir, which may explain why they are more likely to reoccur in the intervention arm.

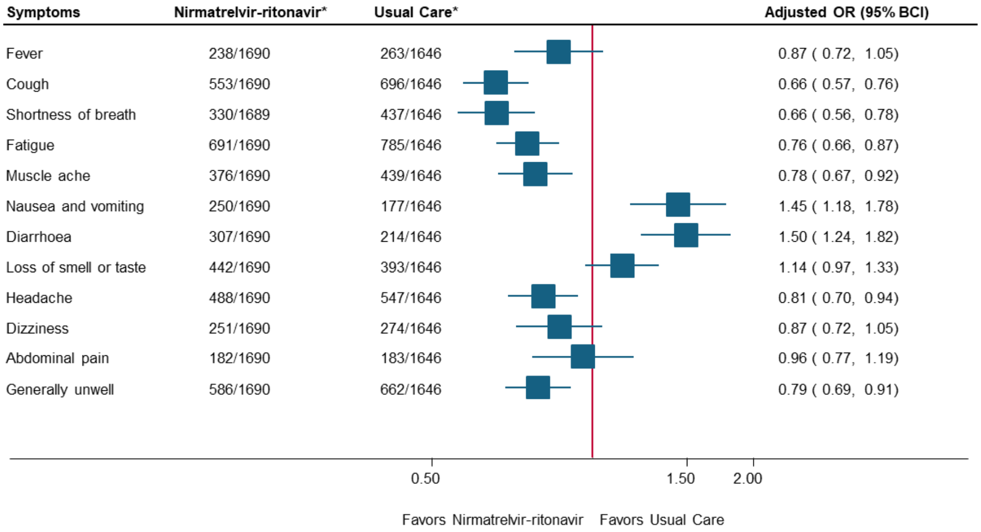

*** Number of recurrent of symptoms by day 28/total reported severe at baseline**

NB: All credible interval widths presented in the graphs have not been adjusted for multiplicity and cannot be used to infer definitive treatment effects.

Subgroup Analysis

Bayesian model estimates and 95% credible intervales presented for the moderation analysis of the primary outcome. An odds ratio<1 favours nirmatrelvir-ritonavir.

Figure S7 Forest plot of Subgroup analysis of primary outcome [PANORAMIC]

Subgroup	Nirmatrelvir-ritonavir	Usual Care	Adjusted Odds ratio (95% BCI)
**Comorbidity**			
No	1/600	2/567	0.55 (0.06, 3.13)
Yes	13/1098	9/1106	1.38 (0.60, 3.30)
**Age**			
<65 years	13/1365	9/1337	1.24 (0.54, 2.85)
≥65 years	1/333	2/336	0.66 (0.05, 5.45)
**Lung disease**			
No	9/1255	6/1268	1.34 (0.52, 3.52)
Yes	5/443	5/405	0.95 (0.28, 3.17)
**Compromised immune system**			
No	13/1553	9/1522	1.29 (0.59, 3.03)
Yes	1/145	2/151	0.57 (0.04, 4.30)
**Obesity**			
No	9/1411	10/1382	0.91 (0.37, 2.25)
Yes	5/287	1/291	3.53 (0.70, 31.91)
**Major symptom**			
No	6/965	3/1001	1.57 (0.49, 5.75)
Yes	8/733	8/672	1.00 (0.37, 2.60)
**PCR confirmed COVID-19**			
No	10/1129	4/1090	1.83 (0.66, 5.52)
Yes	4/569	7/583	0.63 (0.17, 2.02)
**Duration of symptoms**			
≤3 days)	8/1239	7/1227	1.04 (0.40, 2.70)
>3 days	6/459	4/445	1.55 (0.47, 5.60)
**Taking inhaled corticosteroids**			
No	10/1236	6/148	1.45 (0.60, 3.90)
Yes	4/462	5/425	0.81 (0.21, 2.95)

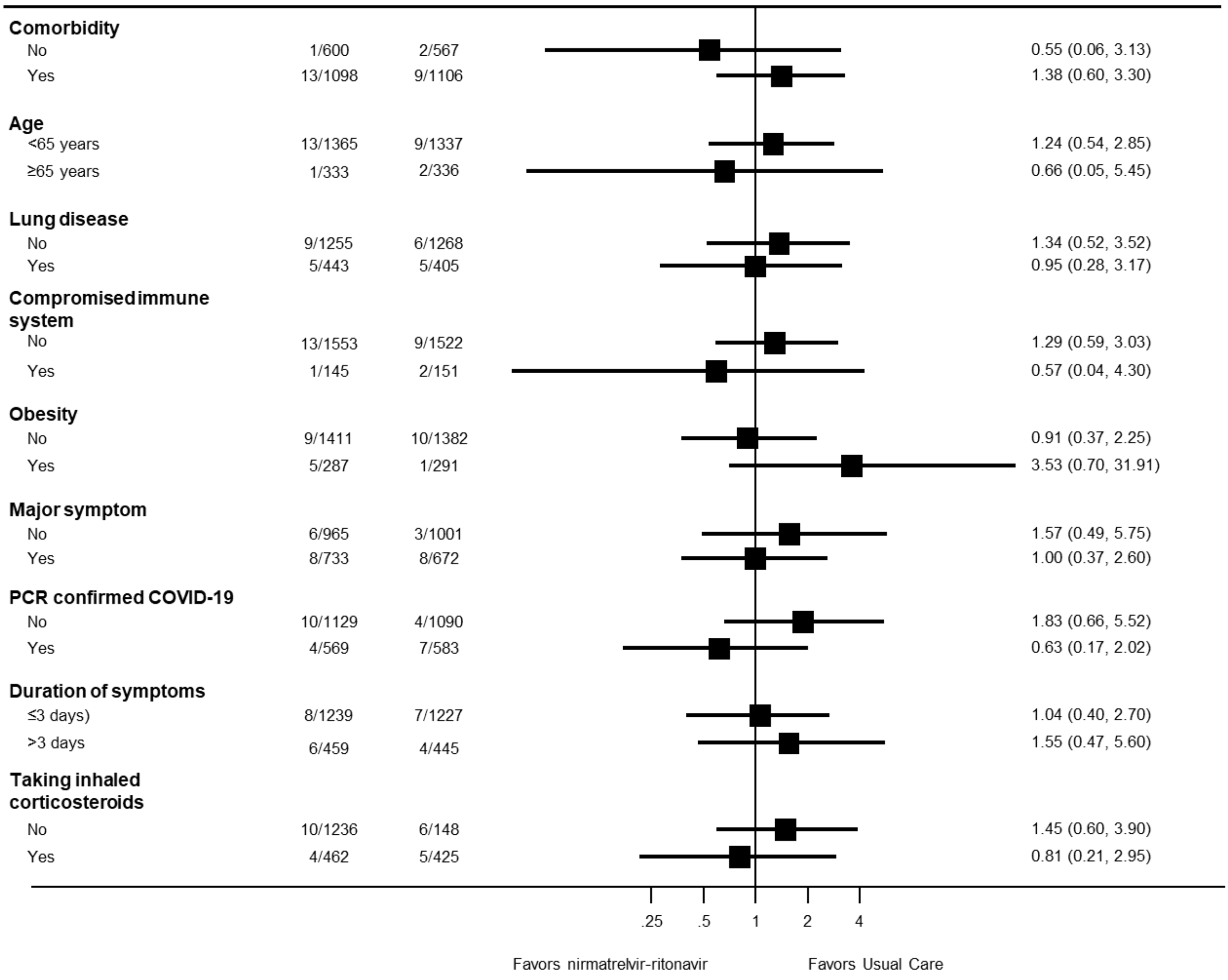

NB: All credible interval widths presented in the graphs have not been adjusted for multiplicity and cannot be used to infer definitive treatment effects

Bayesian model estimates and 95% credible intervales are presented for the moderation analysis of time to recovery. A hazard ratio>1 favours nirmatrelvir-ritonavir.

Figure S8 Forest plot of subgroup analysis of time to first reported recovery [PANORAMIC]

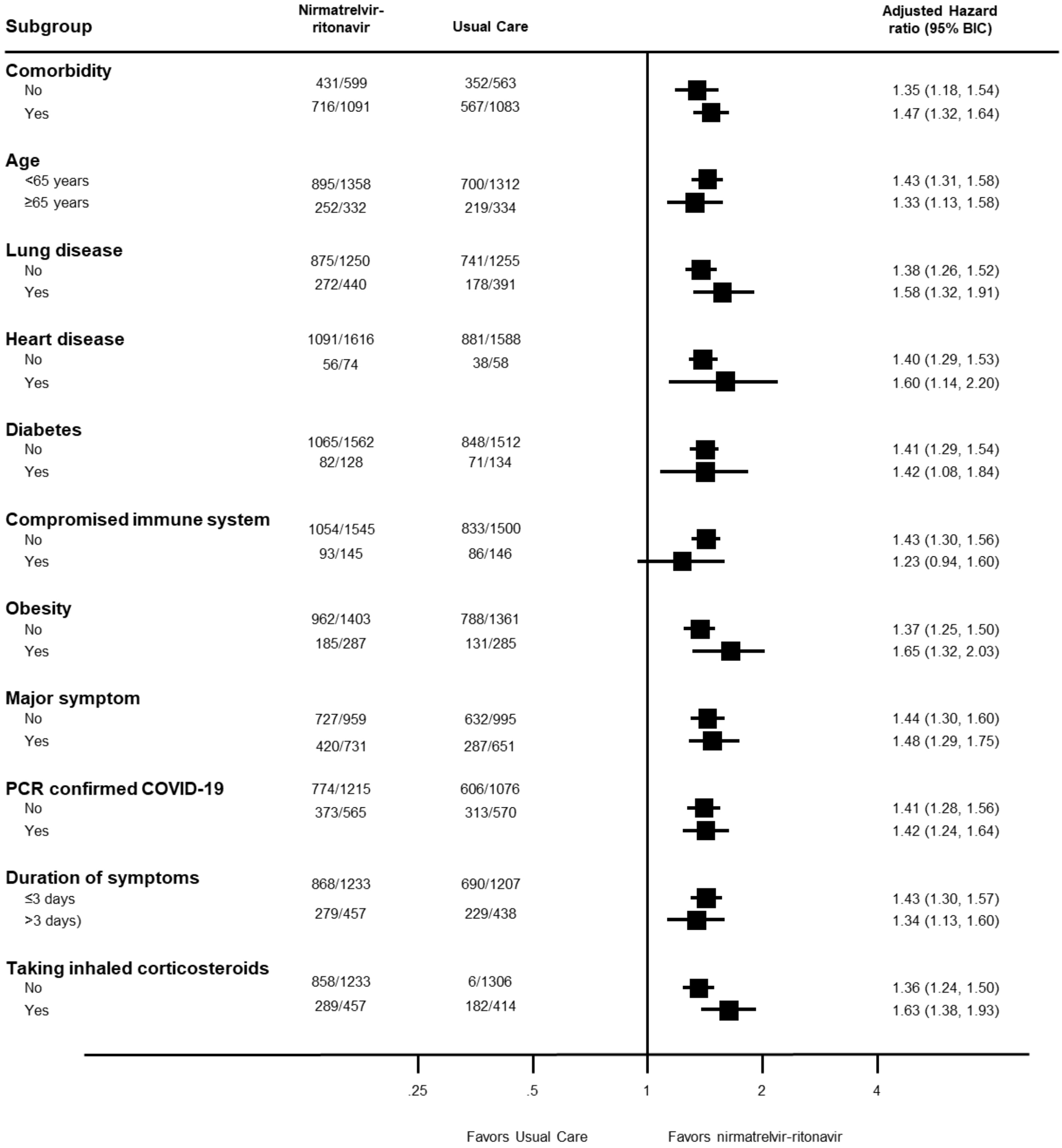

NB: All credible interval widths presented in the graphs have not been adjusted for multiplicity and cannot be used to infer definitive treatment effect.

Sensitivity Analysis

Exploring the assumptions about the prior distribution

Sensitivity analysis of the reported results to assumptions about the prior distributions was assessed for the primary outcome by fitting the maximum likelihood estimates, which produced comparable results.

Table S12 Maximum likelihood model estimates to explore sensitivity to prior distribution [PANORAMIC]

**Outcome**	**Panoramic**	
	**Nirmatrelvir-ritonavir**	**Usual Care**
Hospitalization or death		
Number hospitalized or died	14/1698 (0.8%)	11/1673 (0.7%)
Odds ratio (95% confidence interval)	1.23 (0.55, 2.73)	

NB: All credible interval widths presented in the graphs have not been adjusted for multiplicity and cannot be used to infer definitive treatment effect.

Missing Data

Less than 5% of the data for the primary outcome were missing, so no multiple imputation was carried out as specified in the SAP. However, tipping point analysis was carried out as a post-hoc sensitivity analysis, whereby missing data in the usual care and intervention arms was imputed based on different scenarios to assess how different the unobserved data would have to be to change the analysis results. This was carried out by imputing the outcome to be 1 or 0 based on different event rates in the missing data.

Tipping point analysis was used to investigate the impact of missing data under different scenarios, specifically how different the event rate within the missing data would have to be to change the results. 38/1736 (2.2%) eligible nirmatrelvir-ritonavir participants and 95/1768 (5.4%) of eligible usual care participants had missing primary outcome data. Figure S9 shows results from the tipping point analysis when between 1 and 38 participants were imputed with an event in the usual care group against the 1 to 38 participants in nirmatrelvir-ritonavir were imputed with a primary event. Figure S10 is the corresponding probability of superiority for nirmatrelvir-ritonavir over usual care when the missing data were imputed. To achieve a statistically significant treatment effect favouring nirmatrelvir-ritonavir, an odds ratio of <1 at 0.975, i.e. dark blue region favouring nirmatrelvir-ritonavir in Figure S9 and yellow region in Figure S10, at least 12 missing participants in the usual care and fewer than 2 participants in nirmatrelvir-ritonavir would need to have a primary

outcome event rate. Due to low event rates observed in both groups, it is unlikely the results and conclusion could be tipped over in PANORAMIC.

Figure S9 Heatplot of the odds ratio compared to different numbers of events within the unobserved data [PANORAMIC]

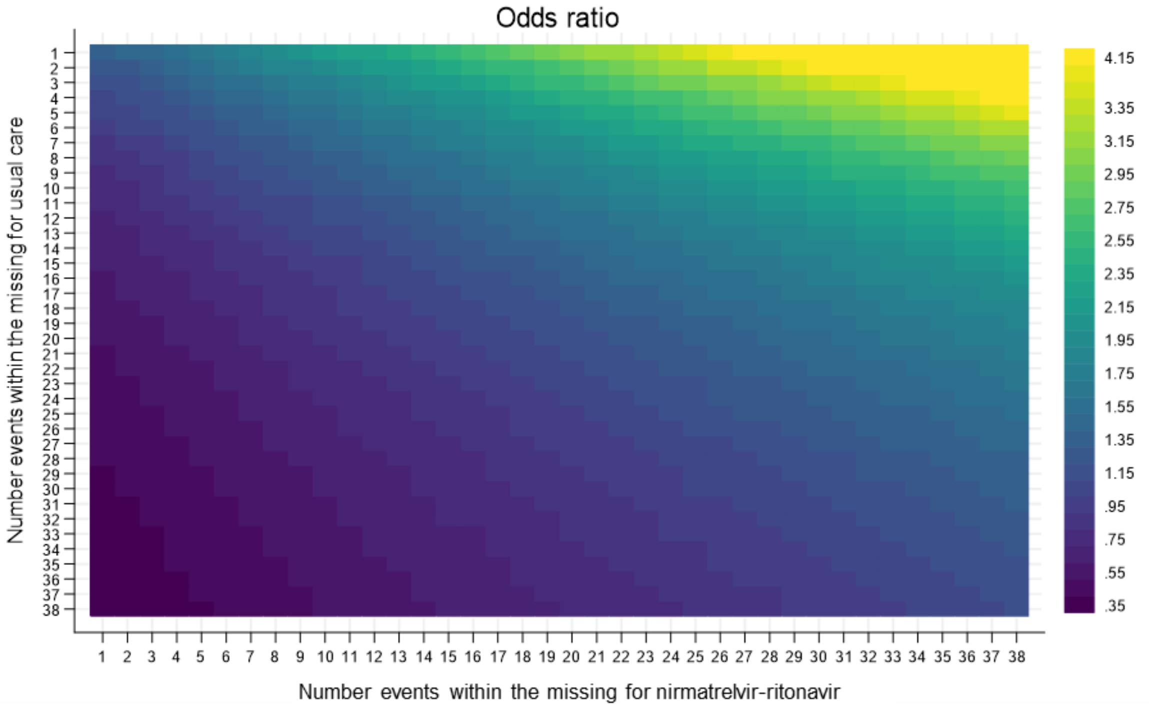

Figure S10 Heatplot of the probability of superiority compared to the number of events within the unobserved data [PANORAMIC]

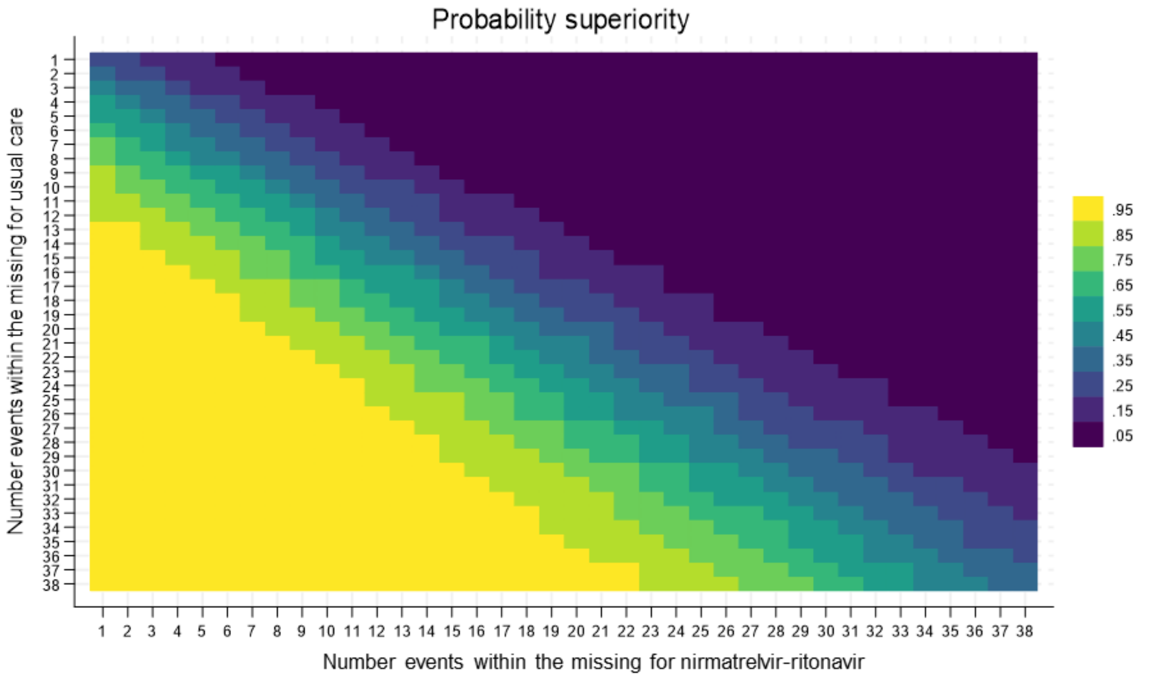

Adverse Events and Serious Adverse Events

Table S13 Adverse events coded by MedDRA Preferred Terms (some AEs have more than one MedDRA codes)* [PANORAMIC]

AE preferred term	N
Dysgeusia	1,059
Taste disorder	439
Diarrhoea	411
Nausea	286
Decreased appetite	279
Abdominal pain	184
Pruritus	134
Headache	116
Chromaturia	92
Faeces pale	88
Dizziness	76
Abdominal pain upper	71
vomiting	58
Fatigue	33
Tinnitus	27
Dyspepsia	20
Dry mouth	19
Insomnia	16
Muscle spasms	16
Oropharyngeal pain	16
Abdominal discomfort	15
Brain fog	15
Cough	15
Dyspnoea	15
Rash	14
Hyperhidrosis	13
Arthralgia	12
Paraesthesia	12
Chest pain	11
Night sweats	11
Rash pruritic	11
Constipation	10
Gastrooesophageal reflux disease	10
Pain in extremity	10
Abdominal distension	9
Feeling abnormal	9
Flatulence	9
Lymphadenopathy	9
Myalgia	9
Abdominal pain lower	8

AE preferred term	N
Anosmia	8
Migraine	8
Palpitations	8
Back pain	7
Epistaxis	7
Lower respiratory tract infection	7
Ocular icterus	7
Pollakiuria	7
Productive cough	7
Eye pruritus	6
Hot flush	6
Nasal congestion	6
Oral herpes	6
Sleep disorder	6
Thirst	6
Tongue coated	6
Ageusia	5
Eye pain	5
Parosmia	5
Renal pain	5
Sensitive skin	5
Vision blurred	5

* Note that only AEs that had at least 5 events are reported here.

Table S14 Adverse events coded by MedDRA System Organ Class (some AEs have more than one MedDRA codes)* [PANORAMIC]

System Organ Class	N
Nervous system disorders	1774
Gastrointestinal disorders	1266
Metabolism and nutrition disorders	287
Skin and subcutaneous tissue disorders	209
Renal and urinary disorders	109
Respiratory, thoracic and mediastinal disorders	86
General disorders and administration site conditions	81
Musculoskeletal and connective tissue disorders	67
Psychiatric disorders	48
Ear and labyrinth disorders	39
Infections and infestations	32
Eye disorders	30
Investigations	14
Cardiac disorders	10
Hepatobiliary disorders	10
Blood and lymphatic system disorders	9
Vascular disorders	9
Reproductive system and breast disorders	6
Injury, poisoning and procedural complications	4
Immune system disorders	1
Product issues	1
Social circumstances	1

Table S15 Serious adverse events coded by MedDRA System Organ Class [PANORAMIC]

System Organ Class	N
Gastrointestinal disorders	3
Respiratory, thoracic and mediastinal disorders	2
Infections and infestations	1
Hepatobiliary disorders	1
Psychiatric disorders	1
Neoplasms benign, malignant and unspecified (incl cysts and polyps)	1

Supplementary Tables and Figures for the CanTreatCOVID study

Definitions of the secondary outcomes reported below and details of analysis can be found in the statistical analysis plan.

Table S16 Secondary outcomes [CanTreatCOVID]

Outcome	Nirmatrelvir-ritonavir	Usual Care	Estimated treatment effect (95% BCI)
**Time to sustained recovery**			
Number recovered by day 14, n/N(%)	223/345 (64.6%)	158/306 (51.6%)	
Median time to recovery, days, median (IQR)	10 (5 to not reached)*	14 (8 to not reached) *	
Hazard ratio (95% credible interval) for each time interval using a time varying piecewise exponential model			
Time interval 1 to 2 days			1.73 (0.80 to 3.93)†
3 to 7 days			1.89 (1.40 to 2.62)†
8 to 11 days			1.14 (0.77 to 1.67)†
12 to 14 days			1.26 (0.82 to 1.96)†
**Time to alleviation of all symptoms**			
Number alleviated by day 14, n/N(%)	273/304 (89.8%)	270/319 (84.6%)	
Median time to alleviation, days, median (IQR)	3 (2 to 6)*	3 (1 to 8)*	1.18 (1.00 to 1.18)‡
**Time to sustained alleviation of all symptoms**			
Number alleviated by day , n/N(%)	240/304 (78.9%)	241/319 (75.5%)	
Median time to alleviation, days, median (IQR)	8 (3 to not reached)*	12 (5 to not reached)*	1.10 (0.93 to 1.30)‡
**Time to Initial reduction of severity of symptoms**			
Number alleviated by day 14, n/N(%)	330/337 (97.2%)	283/295 (95.9%)	
Median time to alleviation, days, median (IQR)	5 (3 to 13)*	6 (3 to not reached)*	0.91 (0.6, 1.56)‡
**Number of days with any severe symptoms, median (IQR) [n]**	3 (1 to 6) [348]	3 (1 to 6) [306]	
**Number of reporting severe symptoms, n/N (%)**			
Day 7	70/257 (27.2%)	101/250 (40.4%)	
Day 14	53/210 (25.2%)	60/202 (29.7%)	

NB: All credible interval widths for the secondary outcomes have not been adjusted for multiplicity and cannot be used to infer definitive treatment effects.
* Kaplan-Meier estimates of median time to event and interquartile range from the raw data.
†Estimated HR derived from a time varying piecewise exponential model adjusted for age, comorbidity and vaccination status. HR > 1 favours Nirmatrelvir-ritonavir
‡ Estimated HR derived from a piecewise exponential model adjusted for age, comorbidity and vaccination status. HR > 1 favours Nirmatrelvir-ritonavir
¶Bayesian logistic regression, adjusted for, age, comorbidity and vaccination status

Health and social care service use

1. Any contact with health care services is defined as reporting use of that service at any point in the daily diary. An odds ratio<1 favours nirmatrelvir-ritonavir
2. Number of contacts with healthcare services is the total count of uses across daily diaries. The median number of contact for each group is reported. A rate ratio<1 favours nirmatrelvir-ritonavir.

Table S17 Self-reported contacts with healthcare services [CanTreatCOVID]

Outcome	Nirmatrelvir-ritonavir	Usual Care	Median Estimate (rate ratio), 95% Bayesian credible interval
Any contact with family doctor	75/349 (21.5%)	68/309 (22.0%)	0.990 (0.677 to 1.419)
Number of contacts with family doctor	101	108	0.835 (0.631 to 1.094)
Any contact with other primary care services (e.g. walk-in clinic)	18/349 (5.2%)	13/309 (4.2%)	1.259 (0.625 to 2.664)
Number of contacts with other primary care services (e.g. walk-in clinic)	22	18	1.105 (0.589 to 2.034)
Any contact with provincial telephone health advice service	19/349 (5.4%)	16/309 (5.2%)	1.062 (0.549 to 2.096)
Number of contacts with provincial telephone health advice service	23	20	1.020 (0.566 to 1.859)
Any contact with emergency department	12/349 (3.4%)	10/309 (5.5%)	1.062 (0.458 to 2.535)
Number of contacts with emergency department	15	17	0.803 (0.407 to 1.632)
Any contact with other healthcare services	39/349 (11.2%)	36/309 (11.7%)	0.961 (0.595 to 1.568)
Number of contacts with other healthcare services	63	59	0.951 (0.670 to 1.377)

NB: All credible interval widths for the outcomes have not been adjusted for multiplicity and cannot be used to infer definitive treatment effects.

Kaplan-Meier Plots for time to event outcomes

Figure S11 Kaplan-Meier curves for time to recovery [CanTreatCOVID]

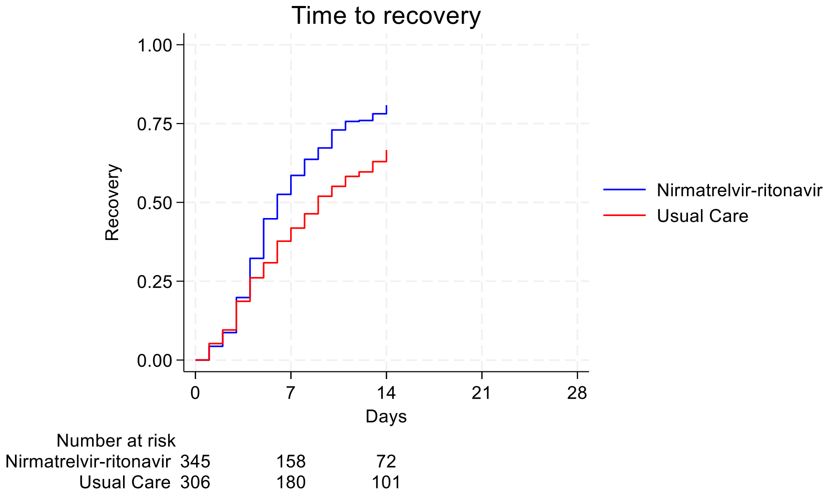

Figure S12 Kaplan-Meier curves for time to sustained recovery [CanTreatCOVID]

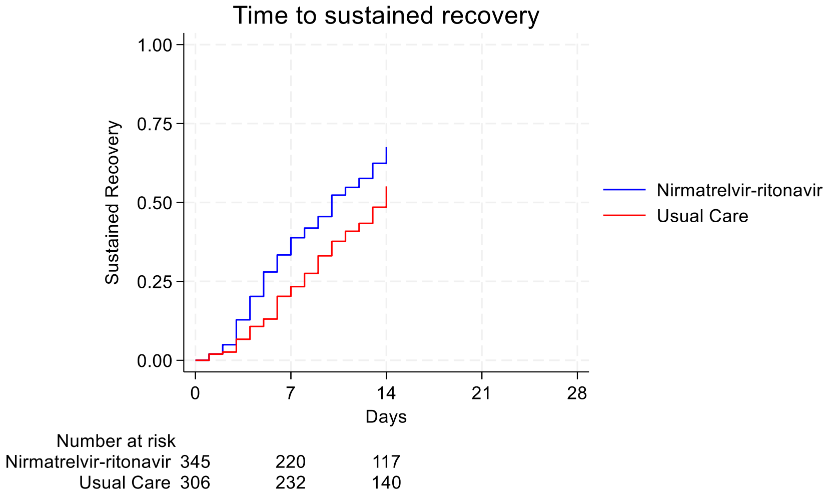

Analysis of Individual Symptoms

The analysis of the CanTreatCOVID dataset includes all data up to day 14.

Figure S13 Time to alleviation of symptoms [CanTreatCOVID]

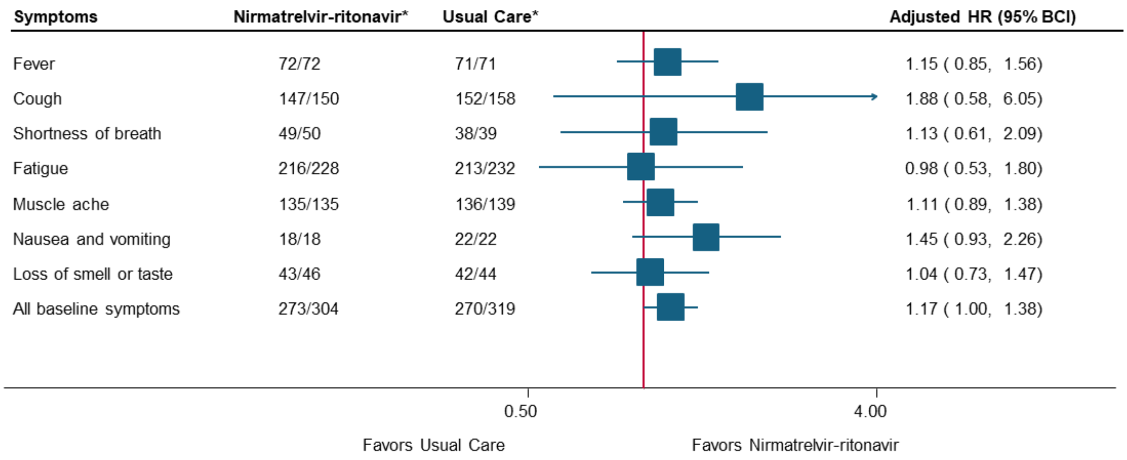

* **Number of alleviation of symptoms by day 14/total reported severe at baseline**

Figure S14 Time to sustained alleviation of symptoms [CanTreatCOVID]

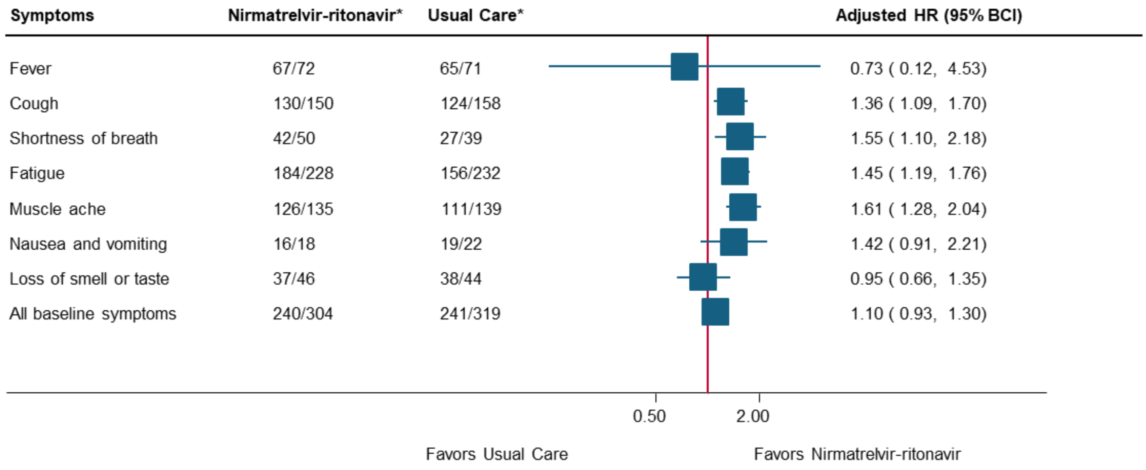

* **Number of sustained alleviation of symptoms by day 14/total reported severe at baseline**

NB: All credible interval widths presented in the graphs have not been adjusted for multiplicity and cannot be used to infer definitive treatment effect.

Figure S15 Time to reduction of symptoms [CanTreatCOVID]

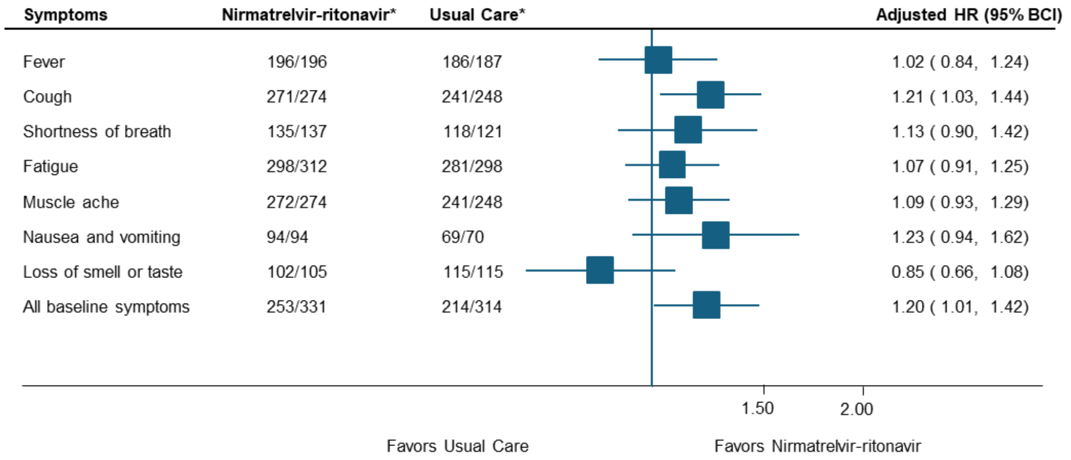

***Number of initial reduction of symptom by day 14/total reported at least mild symptom at baseline. Due to very low event rates the models were not adjusted for vaccination status, as this prevented the MCMC from converging.**

Figure S16 Time to reduction of severity of symptoms [CanTreatCOVID]

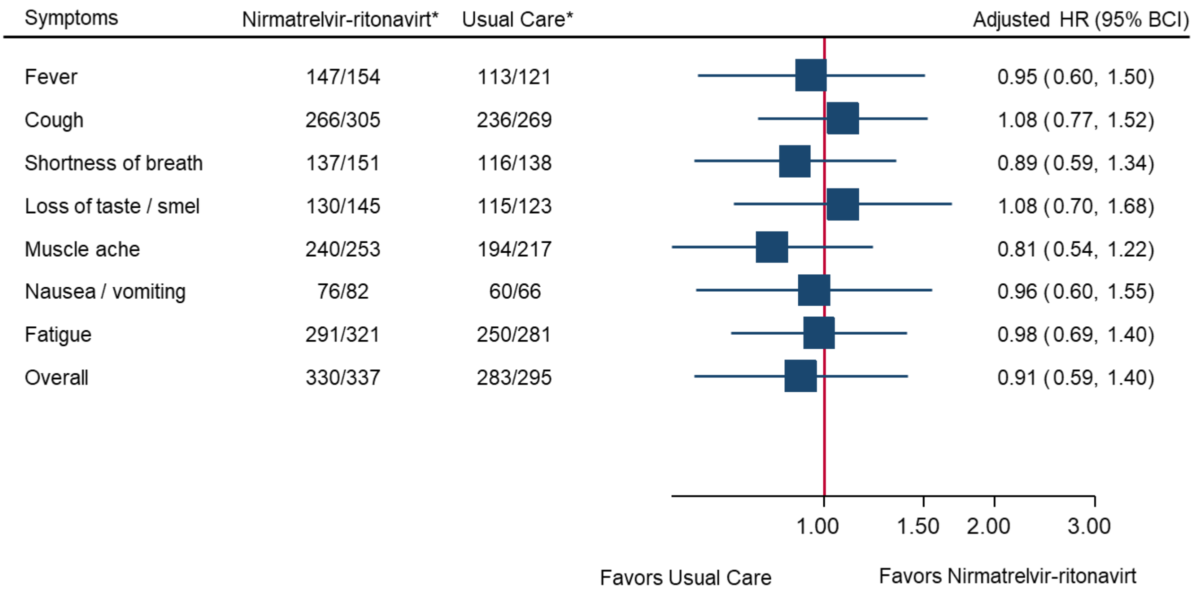

***Number of reduction of symptom by day 14/total reported severe symptom at baseline.**

NB: All credible interval widths presented in the graphs have not been adjusted for multiplicity and cannot be used to infer definitive treatment effect.

Subgroup Analysis

Bayesian model estimates and 95% credible intervales are presented for the moderation analysis of the primary outcome. An odds ratio<1 favours nirmatrelvir-ritonavir.

Figure S17 Forest plot of Subgroup analysis of primary outcome [CanTreatCOVID]

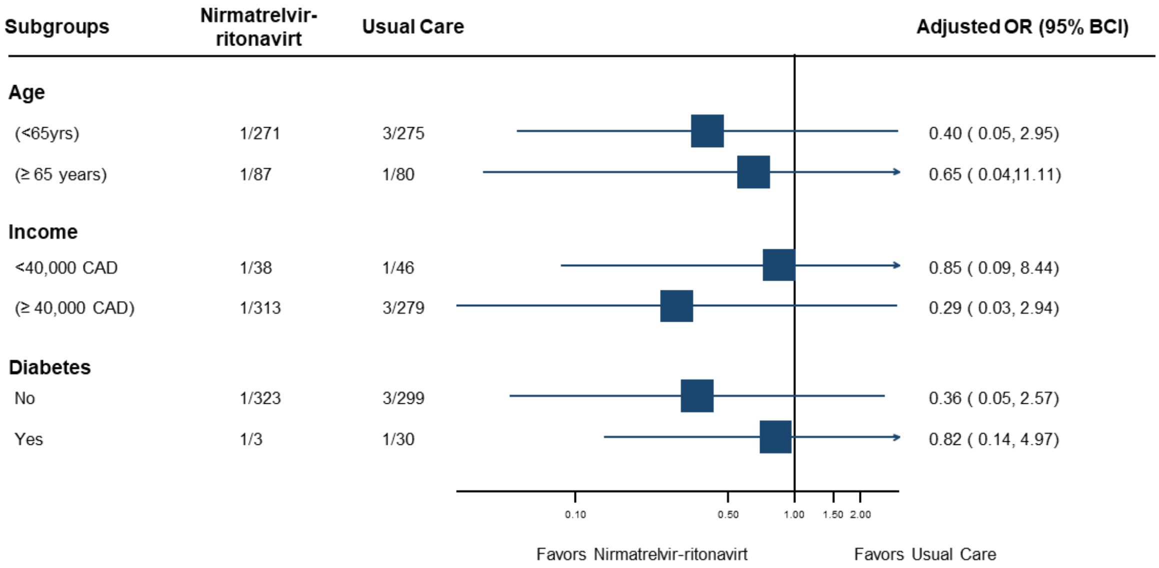

NB: All credible interval widths presented in the graphs have not been adjusted for multiplicity and cannot be used to infer definitive treatment effect.

Bayesian model estimates and 95% credible intervales are presented for the moderation analysis of time to recovery. A hazard ratio>1 favours nirmatrelvir-ritonavir.

Figure S18 Forest plot of subgroup analysis of time to first reported recovery [CanTreatCOVID]

Subgroups	Nirmatrelvir-ritonavirt	Usual Care	Adjusted HR (95% BCI)
**Age**			
<65yrs	200/259	150/239	1.37 (1.13, 1.66)
≥ 65 years	72/86	44/67	1.61 (1.18, 2.20)
**Ethnicity**			
White	223/282	161/255	1.36 (1.13, 1.63)
Black	2/2	3/3	1.50 (0.83, 2.71)
Asian	40/48	18/31	1.80 (1.23, 2.63)
Mixed	4/6	2/5	1.31 (0.76, 2.24)
Other	3/6	10/12	1.11 (0.66, 1.88)
**Income**			
<40,000 CAD	14/40	25/35	1.53 (1.09, 2.15)
≥ 40,000 CAD	178/261	243/304	1.35 (1.12, 1.63)
**Vaccine dose**			
0	2/2	0/2	1.21 (0.81, 1.80)
1	4/6	1/3	1.15 (0.62, 2.13)
2+	266/337	193/301	1.44 (1.20, 1.72)
**Obseity**			
No	223/287	168/251	1.38 (1.15, 1.65)
Yes	49/57	26/55	1.51 (1.03, 2.21)
**Diabetes**			
No	206/259	154/239	1.39 (1.16, 1.67)
Yes	66/85	40/67	1.52 (1.00, 2.31)
**Heart Disease**			
No	267/338	190/300	1.40 (1.17, 1.67)
Yes	5/6	4/6	1.33 (0.79, 2.25)

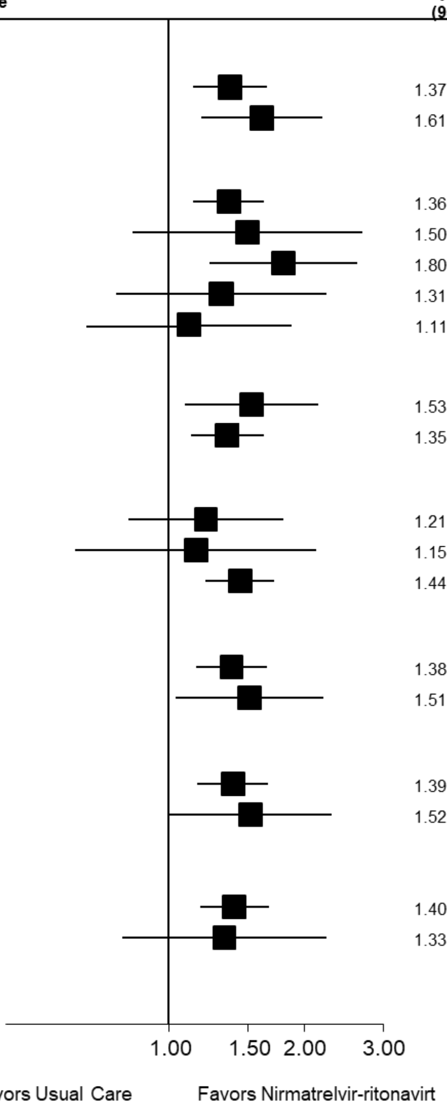

NB: All credible interval widths presented in the graphs have not been adjusted for multiplicity and cannot be used to infer definitive treatment effect.

Sensitivity Analysis

Exploring the assumptions about the prior distribution

Sensitivity analysis of the reported results to assumptions about the prior distributions was assessed for the primary outcome by fitting the maximum likelihood estimates, which produced comparable results.

Table S18 Maximum likelihood model estimates to explore sensitivity to prior distribution [CanTreatCOVID]

Outcome	CanTreatCovid	
	**Nirmatrelvir-ritonavir**	**Usual Care**
Hospitalization or death		
Number hospitalized or died	2/343 (0.6%)	4/324 (1.2%)
Odds ratio (95% confidence interval)	0.47 (0.09 to 2.62)	

NB: All credible interval widths for the outcome has not been adjusted for multiplicity and cannot be used to infer definitive treatment effects.

Missing Data

Multiple imputation model

As there were >5% missing data, multiple imputation was used to assess the sensitivity to the missing at random assumption. The multiple imputation model incorporated baseline age, vaccination status, comorbidities, recovery status at 14 days, and seven baseline symptoms (fever, cough, shortness of breath, fatigue, muscle ache, nausea vomiting, and loss of smell or taste) as predictors. In the dataset, responses for the seven symptoms ranged from “No problem," "Mild problem," "Moderate problem," to "Major problem". For better model convergence, responses of “No problem," "Mild problem," and "Moderate problem" were coded as 0, and "Major problem" as 1. Individual Bayesian models were developed for each treatment group using available data. These models facilitated the generation of samples of outcomes for missing data points from their respective posterior predictive distributions.

The multiple imputation process combined the imputed outcomes with existing complete-case data to produce a full dataset. Each dataset was then analysed using a Bayesian logistic regression model, and the model parameters along with posterior draws were recorded. This procedure was repeated 100 times, creating 100 sets of models and associated

posterior draws. These posterior draws were then combined to compute the final Bayesian credible intervals and the probability of treatment superiority.

The table below summarizes the odds ratio of treatment vs control for the hospitalization endpoint in the multiple imputation analysis. Results were consistent with the primary analysis population when missing data were excluded from the analysis.

Table S19 Multiple imputation results for the primary outcome [CanTreatCOVID]

	Odds ratio (95% BCI)
Hospitalisation/death	0.46 (0.07 to 2.07)

NB: All credible interval widths for the outcome has not been adjusted for multiplicity and cannot be used to infer definitive treatment effects.

Tipping point analysis

Tipping point analysis was used to investigate the impact of missing data under different scenarios, specifically how different the event rate within the missing data would have to be to change the results. 15/358 (4.2%) eligible nirmatrelvir-ritonavir participants and 34/358 (9.5%) of eligible usual care participants had missing primary outcome data. Figure S19 shows results from the tipping point analysis when between 1 and 34 participants were imputed with an event in the usual care group against the 1 to 15 participants in nirmatrelvir-ritonavir were imputed with a primary event. Figure S20 is the corresponding probability of superiority for nirmatrelvir-ritonavir over usual care when the missing data were imputed. To achieve a statistically significant treatment effect favouring nirmatrelvir-ritonavir, an odds ratio of <1 at 0.975, i.e. dark blue region favouring nirmatrelvir-ritonavir in Figure S19 and yellow region in Figure S20, at least 2-3 missing participants in the usual care and fewer than 1 participants in nirmatrelvir-ritonavir would need to have a primary outcome event rate. This represents ~6% event rate in the missing data (and almost doubling the event rates in the usual care group), which is more than the 1% in the observed. It is unlikely the results and conclusion could be tipped over in CanTreatCOVID.

Post-hoc sensitivity analysis for cross-over participants

Give that there were 11 participants who were allocated to the usual care but received nirmatrelvir-ritonavir, we carried out post-hoc analyses to assess the impact this may have on the primary outcome.

Table S20 Summary of sensitivity analysis of primary outcome [CanTreatCOVID]

	Nirmatrelvir-ritonavir	Usual Care	Adjusted OR (95% CI)
Cross-overs excluded from usual care	2/343 (0.6%)	4/313 (1.3%)	0.46 (0.08 to 2.52)
Analysed as “treatment receive”	2/354 (0.6%)	4/313 (1.3%)	0.44 (0.08 to 2.44)

NB: All credible interval widths for the outcome has not been adjusted for multiplicity and cannot be used to infer definitive treatment effects.

Figure S19 Heatplot of the odds ratio compared to different numbers of events within the unobserved data [CanTreatCOVID]

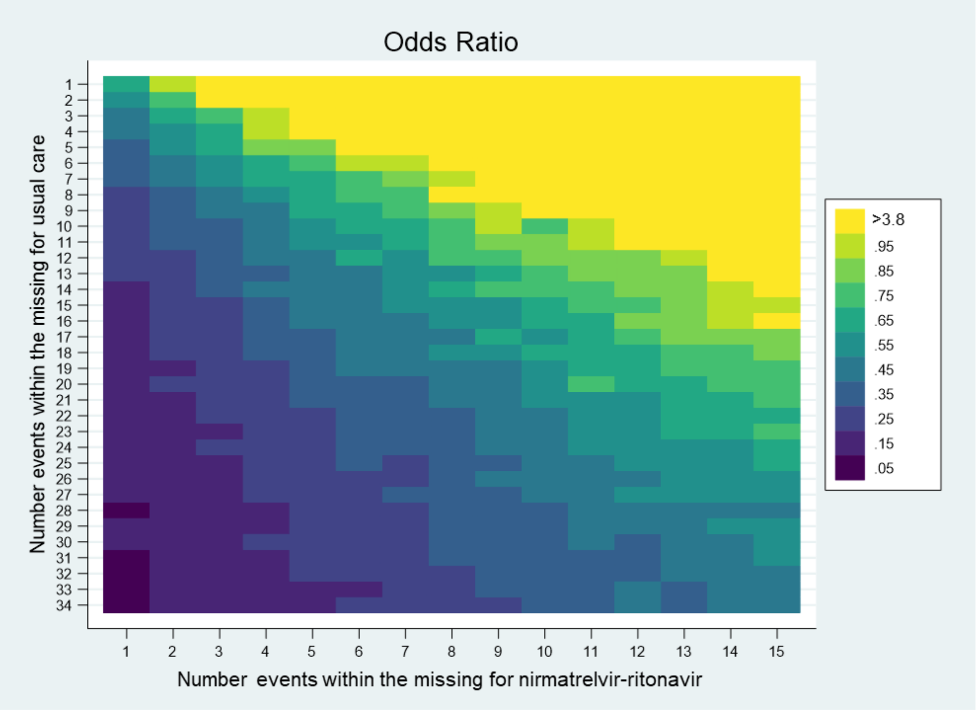

Figure S20 Heatplot of the probability of superiority compared to the number of events within the unobserved data

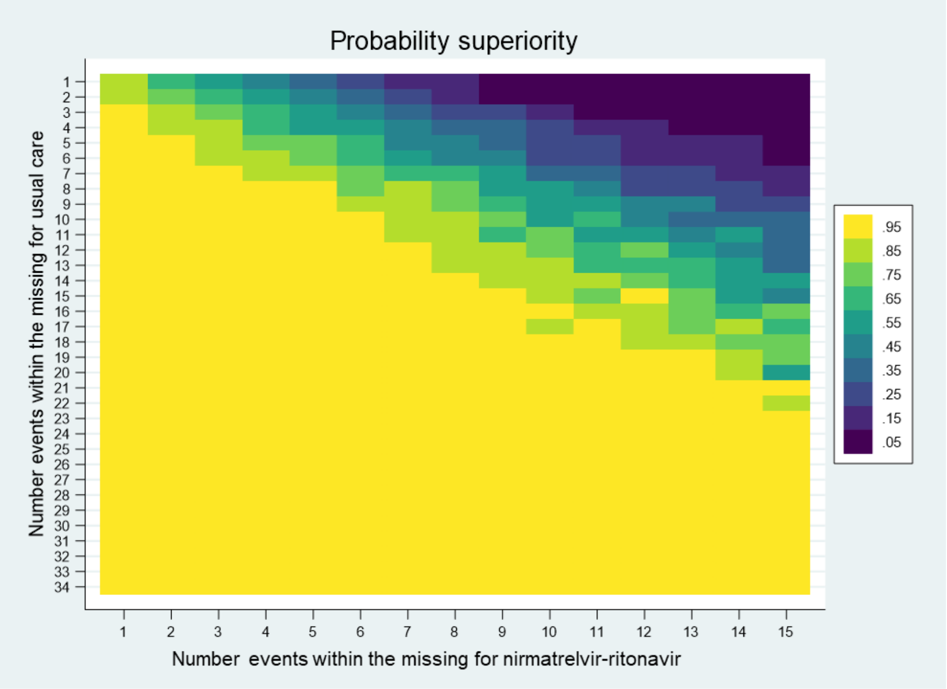

Adverse Events and Serious Adverse Events

Table S21 Adverse events coded by MedDRA Preferred Terms (some AEs have more than one MedDRA codes) [CanTreatCOVID]

In CanTreatCOVID, there are 228 non-serious adverse events in total (affecting 132/716 participates). No participate withdrew due to adverse events. The top three adverse events are Dysgeusia, Diarrhoea and Nausea.

Preferred Term	N
Dysgeusia	47
Diarrhoea	28
Nausea	22
Product after taste	20
Taste disorder	8
Rash	5
Abdominal discomfort	5
Vomiting	3
Abdominal pain upper	3
Headache	3
COVID-19	2
Diverticulitis	2
Pyrexia	2
Migraine	2
Sinusitis	2
Dyspepsia	2
Cough	2
Heart rate increased	2
Urinary tract infection	1
Chest discomfort	1
Bronchitis	1
Cystitis	1
Concussion	1
Cystocele	1
Mole excision	1
Cystocele repair	1
Parotid gland enlargement	1
Dehydration	1
Renal pain	1
Anosmia	1
Tongue coated	1
Disorientation	1
Lower respiratory tract infection	1
Appendicitis	1
Myocardial infarction	1
Dizziness	1
Oral infection	1

Preferred Term	N
Dry eye	1
Poor quality sleep	1
Dupuytren's contracture operation	1
Radius fracture	1
Asthenia	1
Rheumatoid arthritis	1
Asthma	1
Tachycardia	1
Ear infection	1
Upper-airway cough syndrome	1
Faeces pale	1
Viral infection	1
Fatigue	1
Breast cellulitis	1
Femur fracture	1
Muscle spasms	1
Foot fracture	1
Nasal congestion	1
Gastric ulcer haemorrhage	1
Nephrolithiasis	1
Generalised tonic-clonic seizure	1
Pain	1
Gingivitis	1
Pneumonia	1
Haemoptsis	1
Postoperative wound infection	1
Blood cholesterol increased	1
Chromaturia	1
Viral load abnormal	1
Chronic sinusitis	1
Blood triglycerides increased	1
Respiration abnormal	1
Blood urine present	1
Sialoadenitis	1
Hypertension	1
Syncope	1
Incisional hernia repair	1

Preferred Term	N
Abdominal pain	1
Influenza like illness	1
Trigger finger	1
Inguinal hernia repair	1
Urinary tract discomfort	1
Insomnia	1

Preferred Term	N
Vaginal prolapse	1
Joint swelling	1
Ageusia	1
Ligament sprain	1
Heart rate irregular	1
Hip arthroplasty	1

Table S22 Adverse events coded by MedDRA System Organ Class (some AEs have more than one MedDRA codes) [CanTreatCOVID]

System Organ Class	N
Gastrointestinal disorders	68
Nervous system disorder	35
Nervous system disorders	28
Product issues	20
Infections and infestations	20
Respiratory, thoracic and mediastinal disorders	8
Investigations	7
Surgical and medical procedures	6
General disorders and administration site conditions	6
Skin and subcutaneous tissue disorders	5
Injury, poisoning and procedural complications	5
Musculoskeletal and connective tissue disorders	4
Renal and urinary disorders	4
Psychiatric disorders	3
Reproductive system and breast disorders	2
Cardiac disorders	2
Nervous system disorders	2
Metabolism and nutrition disorders	1
Vascular disorders	1
Eye disorders	1

Table S23 Serious adverse events coded by MedDRA System Organ Class [CanTreatCOVID]

System Organ Class	N
Infections and infestations	6
Surgical and medical procedures	6
Nervous system disorders	2
Gastrointestinal disorders	2
General disorders and administration site conditions	2
Injury, poisoning and procedural complications	2
Nervous system disorders	1
Investigations	1
Cardiac disorders	1
